# Primitive Extracellular Lipid Components on the Surface of the Charophytic Alga *Klebsormidium flaccidum* and Their Possible Biosynthetic Pathways as Deduced from the Genome Sequence

**DOI:** 10.3389/fpls.2016.00952

**Published:** 2016-06-30

**Authors:** Satoshi Kondo, Koichi Hori, Yuko Sasaki-Sekimoto, Atsuko Kobayashi, Tsubasa Kato, Naoko Yuno-Ohta, Takashi Nobusawa, Kinuka Ohtaka, Mie Shimojima, Hiroyuki Ohta

**Affiliations:** ^1^Graduate School of Bioscience and Biotechnology, Tokyo Institute of TechnologyKanagawa, Japan; ^2^School of Life Science and Technology, Tokyo Institute of TechnologyKanagawa, Japan; ^3^Core Research for Evolutional Science and Technology, Japan Science and Technology AgencyTokyo, Japan; ^4^The Earth-Life Science Institute, Tokyo Institute of TechnologyTokyo, Japan; ^5^Advanced Course of Food and Nutrition, Nihon University Junior CollegeShizuoka, Japan

**Keywords:** *Klebsormidium flaccidum*, *Chlamydomonas reinhardtii*, *Arabidopsis thaliana*, extracellular lipids, polyester backbone, wax, cuticle

## Abstract

*Klebsormidium flaccidum* is a charophytic alga living in terrestrial and semiaquatic environments. *K. flaccidum* grows in various habitats, such as low-temperature areas and under desiccated conditions, because of its ability to tolerate harsh environments. Wax and cuticle polymers that contribute to the cuticle layer of plants are important for the survival of land plants, as they protect against those harsh environmental conditions and were probably critical for the transition from aquatic microorganism to land plants. Bryophytes, non-vascular land plants, have similar, but simpler, extracellular waxes and polyester backbones than those of vascular plants. The presence of waxes in terrestrial algae, especially in charophytes, which are the closest algae to land plants, could provide clues in elucidating the mechanism of land colonization by plants. Here, we compared genes involved in the lipid biosynthetic pathways of *Arabidopsis thaliana* to the *K. flaccidum* and the *Chlamydomonas reinhardtii* genomes, and identified wax-related genes in both algae. A simple and easy extraction method was developed for the recovery of the surface lipids from *K. flaccidum* and *C. reinhardtii*. Although these algae have wax components, their surface lipids were largely different from those of land plants. We also investigated aliphatic substances in the cell wall fraction of *K. flaccidum* and *C. reinhardtii*. Many of the fatty acids were determined to be lipophilic monomers in *K. flaccidum*, and a Fourier transform infrared spectroscopic analysis revealed that their possible binding mode was distinct from that of *A. thaliana*. Thus, we propose that *K. flaccidum* has a cuticle-like hydrophobic layer composed of lipids and glycoproteins, with a different composition from the cutin polymer typically found in land plant cuticles.

## Introduction

The aerial epidermis of land plants is covered by a cuticle layer that has critical roles in protecting the plants from ultraviolet irradiation (Barnes et al., 1998), in controlling non-stomatal water loss (Riederer and Schreiber, 2001), and in guarding plants from pathogens and insect herbivores (Müler and Riederer, 2005; Skamnioti and Gurr, 2007), as well as in preventing organ fusion during development (Lolle et al., 1998; Sieber et al., 2000). The cuticle in higher plants is composed of three layers, the cuticular layer, which is anchored to the cell wall by oligosaccharides; the cuticle proper, consisting of intracuticular waxes located above the cuticular layer; and the outermost epicuticular wax (Pollard et al., 2008).

Most research on the molecular components of the epicuticular wax has been undertaken using *Arabidopsis thaliana*. Alkanes, ketones, aldehydes, primary and secondary alcohols, and very-long-chain fatty acids are included in wax (Kunst and Samuels, 2003; Samuels et al., 2008; Lee and Suh, 2015). These lipid classes are made up of very-long-chain fatty acyl moieties with more than 20 carbon atoms. Deficiencies in wax components leads to detrimental phenotypes: *eceriferum1-1* (*cer1-1*), a mutant of cuticular wax synthesis, reduces alkane coverage (Bernard et al., 2012) and pollen fertility under low-humidity conditions (Aarts et al., 1995); the loss of CUTICULAR1 activity results in dysfunctional pollen grains (Millar et al., 1999); and *cer7-3* results in reduced seed viability (Hooker et al., 2007). Thus, wax plays various important roles in *A. thaliana*. Epicuticular wax has also been analyzed from Bryophytes, such as *Andreaea* and *Pogonatum* species (Haas, 1982). In this report, the outer lipids of these Bryophytes are composed of alkanes, wax esters, aldehydes, primary alcohols, and fatty acids.

Constituents of the cuticle, including the cuticle proper and cuticular layer, were also investigated in *A. thaliana* (Graça et al., 2002; Franke et al., 2005; Molina et al., 2006). The cuticle of shoots is composed of aliphatic polymers, such as cutin and cutan. The former can be depolymerized by acid or alkaline hydrolysis, whereas the latter is difficult to break down using either acidic or alkaline reagents. Cutin obtained from the hydrolysis of delipidated *Arabidopsis* leaves or stems has been analyzed, and various fatty acid derivatives, such as ω-hydroxy fatty acids, α,ω-dicarboxylic acids, polyhydroxy fatty acids, and glycerol, were found. These monomers form an aliphatic network via primary or secondary ester bonds, producing vigorous structures and interspaces filled with waxes on the plant surface. A null mutant of long-chain acyl-CoA synthetase2 (LACS2) exhibits a decrease in the cutin layer thickness on the abaxial surface of its leaves, and its growth is inhibited in *A. thaliana* (Schnurr et al., 2004). Furthermore, *Oryza sativa* that lacks the cytochrome P450 superfamily gene *CYP704B2* displays reduced growth, male sterility, aborted pollen grains and undeveloped anther cuticles (Li et al., 2010). The *desperado-3* (*dso-3*) mutant in *A. thaliana*, which lacks the ABCG transporter gene, displays a reduced amount of both wax and cutin monomers. Root branching and growth inhibition have also been observed in *dso-3* (Panikashvili et al., 2007).

As lipophilic outer layers are necessary for land plants, it is of great interest to determine whether terrestrial algae also have these outer lipids. Rindi et al. (2008) analyzed *Klebsormidium* species and speculated that some of them have an outer layer, called the “superficial hydro-repellent layer,” which is formed in a liquid medium. However, there is no chemical evidence supporting the presence of a superficial hydro-repellent layer in some microalgae.

The genus *Klebsormidium* belongs to the Klebsormidiales, one of the basal groups of the Charophycean Green Algae (CGA), the extant algal group as a model of ancestors of land plants, that typically have multicellular and non-branching filaments without differentiated cells (Hori et al., 2014). It is one of the most common genera of terrestrial algae and includes a few species of green algae that occur in subaerial and semiaquatic environments worldwide, such as the northernmost habitat of Ny-Ålesund, Norway (Kaštovská et al., 2005), southern Africa (Karsten et al., 2015), the European Alps (Karsten et al., 2010), and Japan. The survival of these algae may be attributed to their high tolerance to desiccation (Herburger and Holzinger, 2015; Karsten et al., 2015), low temperature (Nagao et al., 2008), and osmotic stress (Kaplan et al., 2012).

It is generally accepted that the ancestor(s) of land plants was closely related to modern charophytes (Lewis and McCourt, 2004; Becker and Marin, 2009; Popper et al., 2011; Leliaert et al., 2012; Timme et al., 2012). Initial land colonization has been proposed to be carried out by aquatic algae adapting to a terrestrial environment. Therefore, *Klebsormidium* is an important genus for evolutionary studies. Recently, the draft genome sequence of *Klebsormidium flaccidum* strain NIES-2285 was completed (Hori et al., 2014). Thus, investigating the extracellular lipid components of this alga could be helpful for elucidating details of plant terrestrialization.

Here, we identified the genes encoded by the *K. flaccidum* genome that are homologous to those involved in the wax and cutin synthesis pathways of *A. thaliana*. Because genes related to lipid synthesis were found, we tried to confirm the existence of the extracellular lipids on the surface of *K. flaccidum* using a simple and easy extraction method and analyzed those that were present. In addition, we studied the contents and binding patterns of cutin monomers using gas chromatography–mass spectrometry (GC-MS) and attenuated total reflectance (ATR) Fourier transform infrared spectroscopy (FTIR). Our results demonstrated that the components of the extracellular lipids of *K. flaccidum*, dominated by alkanes and triacylglycerols (TAGs), were similar to those of *Chlamydomonas reinhardtii*, which belongs to the Chlorophyceae, and different from those of *A. thaliana*. *K. flaccidum* likely does not have aliphatic polymers on its cell walls, unlike *A. thaliana*; however, a great amount of fatty acids was covalently attached to the delipidated cell wall fraction, providing a primitive cuticle layer–like structure that may be filled with surface lipids, such as alkanes and TAGs.

## Materials and Methods

### Plant Materials

*Arabidopsis thaliana* ecotype Columbia was grown in soil under continuous light (40–50 μmol photons m^-2^ s^-1^) at 22°C for 42 days. *K. flaccidum* strain NIES-2285 was cultured on a nitrocellulose membrane placed on an agar plate of NIES-C medium (Ichimura, 1971) in a Petri dish under continuous light (10 μmol photons m^-2^ s^-1^) at 23°C for 28 or 42 days. *C. reinhardtii* was cultured on a nitrocellulose membrane placed on an agar plate containing Tris-acetate-phosphate medium under the same conditions as *K. flaccidum* for 14 days. Both nitrocellulose membranes and agar powder were delipidated three times with more than 15 fold amount by weight of chloroform before the cultivation of algae.

### Reagents

Phytol, stigmasterol, and β-sitosterol containing campesterol and the fatty acids including linoleic and linolenic acids, myristoyl, palmitoyl, stearoyl, oleoyl chlorides, and N, O-bis (trimethylsilyl) trifluoroacetamide with 1% trimethylchlorosilane (BSTFA-TMCS, for the trimethylsilyl derivatization) were purchased from Tokyo Chemical Industry Co, Ltd., (TCI). Primuline (for lipid detection) and other organic solvents were purchased from Wako Pure Chemical Industries, Ltd., and silica gel 60 thin-layer chromatography (TLC) plates were purchased from Merck Millipore.

### Synthesis of Steryl and Phytyl Esters

Steryl and phytyl esters carrying specific fatty acids were synthesized by reacting fatty acyl chlorides with sterols or phytol, respectively (Wisnieski et al., 1973; Terzaghi, 1986). Fatty acyl chlorides were prepared by reacting linoleic or linolenic acid as a free fatty acid with an excess of oxalyl chloride in the presence of sodium carbonate (2 mol per mol of fatty acid) in dichloromethane. The reaction mixture was stirred for 6 h at room temperature and then heated for 60 min under reflux at 80°C. Excess oxalyl chloride was removed under vacuum, and then the acyl chloride was dissolved in dichloromethane and added to an equimolar quantity of campesterol plus sitosterol, stigmasterol, or phytol dissolved in dichloromethane with sodium carbonate (2 mol per mol of fatty acid). Pyridine (0.1 ml g acyl chloride^-1^) was added dropwise, and the mixture was stirred for 12 h at room temperature. After the reaction mixture was filtered through sintered glass, the solvent was removed using a rotary evaporator, and steryl or phytyl esters were purified by silica gel TLC with hexane/diethyl ether/acetic acid (80:20:1, v/v/v).

### Scanning Electron Microscopy (SEM)

*Klebsormidium flaccidum* was transferred to a fresh agar medium or liquid medium. Three days after subculturing, algal cells were collected on 1.5% (w/v) agar medium and fixed with 2% (v/v) glutaraldehyde in 0.1 M phosphate buffer. Samples were dehydrated in a graded series of ethanol and t-butyl alcohol, and then dried by using a vacuum evaporator (VFD-21S, Vacuum Device Inc.). The samples mounted on an aluminum stub were sputter-coated with osmium using an osmium coater (Neoc-Pro, Meiwafosis). SEM observations were performed using an S-3400 microscope (Hitachi) at 10 kV.

### Extracellular Lipid Extraction

#### Chloroform Extraction of Extracellular Lipids

**FIGURE 1:**
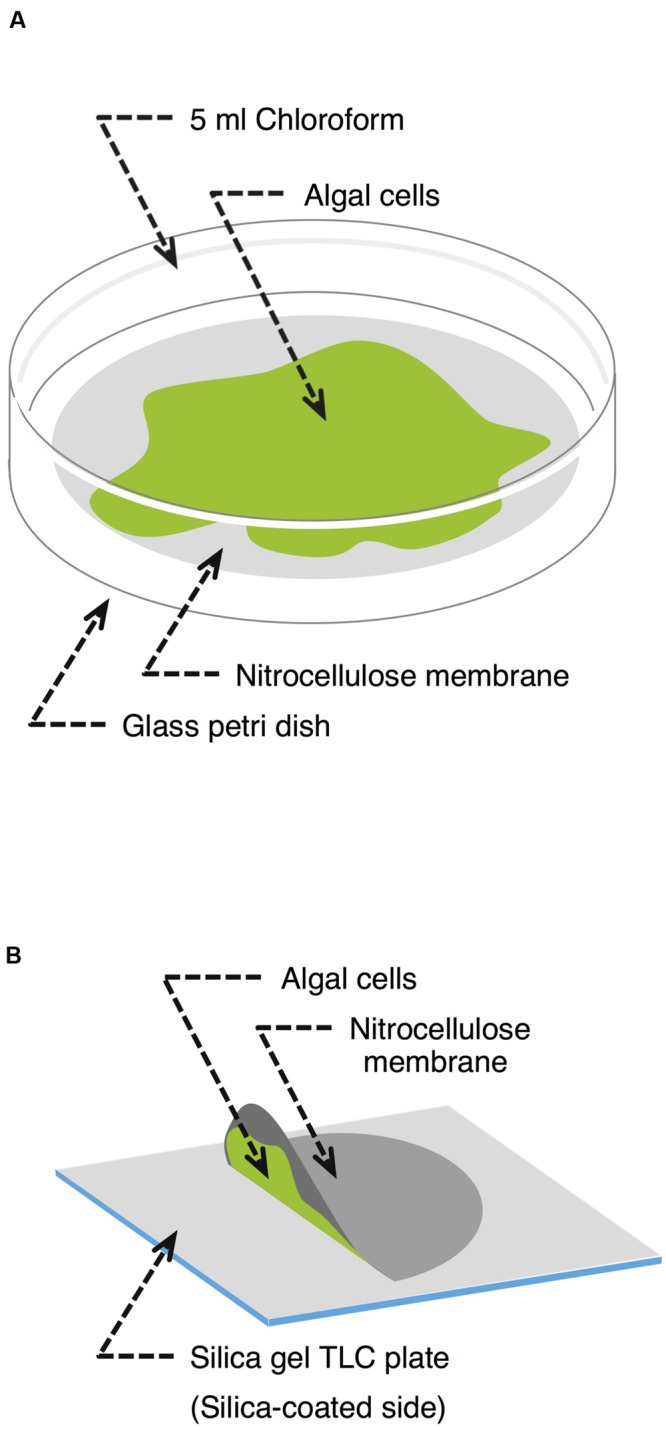
**Schematic representation of the lipid extraction methodologies used. (A)** Chloroform extraction. Alga grown on a nitrocellulose membrane was transferred to a glass Petri dish containing 5 ml chloroform. After a predefined time, the chloroform was recovered and the algal cells were harvested to determine their dry weight. **(B)** Silica gel plate extraction. A nitrocellulose membrane covered with algal cells was placed on a silica gel plate culture facing down without any force for a predetermined period.

*Klebsormidium flaccidum* was cultured for 28 days or *C. reinhardtii* was cultured for 14 days on a nitrocellulose membrane placed on an agar plate. First, to determine an appropriate extraction time to isolate lipids, we immersed the entire membrane with *K. flaccidum* in 5 ml chloroform at room temperature in a glass Petri dish (**Figure 1A**). The solvent was recovered at predetermined time intervals of 10, 20, 30, 60, 120, 240, 480, and 1920 s. At each sampling time, the solvent was retrieved and then 5 ml of fresh chloroform was immediately added to the Petri dish for the next time point. The chloroform extracts were filtered and combined from 2 cultures, and then the solvent was removed with a gentle stream of nitrogen while heating to 30°C. The remaining lipids were weighed and subsequently dissolved in chloroform at 20 mg lipids ml^-1^. After the final extraction, a portion of alga was dried for measuring the weight, and total lipids were extracted from aliquot as described by Bligh and Dyer (Bligh and Dyer, 1959) to determine the amounts of triacylglycerol (TAG) and monogalactosyldiacylglycerol (MGDG). As the protocol for chemical analysis, 30 s was employed as the extraction time, and the extracellular lipids were collected from 7 cultures.

#### Silica Gel Plate Extraction of Extracellular Lipids

*Klebsormidium flaccidum* was grown on nitrocellulose membranes as described above but for 42 days. Each membrane on which *K. flaccidum* cells were growing was then placed growing surface down on a silica gel 60 TLC plate (Merck Millipore) without any force for 30 s, 1, 2.5, 5, 10, and 20 min (**Figure 1B**), during which time point the extracellular lipids secreted from cells were transferred to the plate. After the membrane with the cells was carefully removed, the silica gel plate was dried in a fume hood at room temperature for 30 min. Lipids were detected using primuline under ultraviolet light (see below) and recovered from the silica gel plate by scraping the surface lipids into chloroform. The same process was used with *C. reinhardtii* after subculturing for 14 days. Wax was also extracted from *A. thaliana* by pressing it to a silica gel plate at approximate 100 g cm^-1^ by hand.

#### Extracellular Lipid Separation and Derivatization

The lipids were separated by TLC before the GC-MS analysis. The extracts were applied to silica gel 60 TLC plates and developed using the following solvent systems sequentially: (1) to a height of 20 cm with hexane; (2) to a height of 20 cm with toluene; and, (3) to a height of 10 cm with hexane/diethyl ether/acetic acid (70:30:1, v/v/v) (Yamashiro et al., 2001). To determine the extraction time, TAG and MGDG were separated on a TLC plate by using the following sequential two-part development system: (1) to a height of 20 cm with hexane/diethyl ether/acetic acid (70:30:1, v/v/v) and, (2) to a height of 10 cm with acetone/toluene/water (91:30:8, v/v/v). All of the lipids on the TLC plates were visualized under ultraviolet light after the plates were sprayed with 0.01% (w/v) primuline in 80% (v/v) acetone. The sterol lipid fraction was derivatized with 20 μl BSTFA-TMCS and 20 μl pyridine for 60 min at 80°C to transform all of the hydroxyl-containing groups into their corresponding trimethylsilyl derivatives. Fatty acid methyl esters were obtained from the TAG fraction by methanolysis with 350 μl of 5% hydrochloric acid in methanol for 60 min at 80°C in a screw-capped test tube. The lipids corresponding to steryl esters were hydrolyzed by 1 ml of 2 M sodium methoxide solution in methanol at 80°C for 60 min. The resultant fatty acids and sterols were derivatized by 20 μl BSTFA-TMCS and 20 μl pyridine for 60 min at 70°C. Alkanes and phytyl esters were analyzed without derivatization. For all of the classes, the lipids were characterized by GC-MS and quantified by GC-flame ionization detection (FID).

#### Lipid Characterization by GC-MS and GC-FID

The ratios of the lipids that corresponded to steryl esters, wax esters, and sterols were further identified using capillary gas chromatography (GC) connected to a electron ionization mass spectrometric detector (EI-MS; GC-2010 equipped with GCMS-TQ8030, Shimadzu) with a 30-m DB-5 column (0.25-mm i.d.; film thickness, 0.25 μm; Agilent) with He. The carrier gas inlet pressure was regulated for a constant flow of 1.4 ml min^-1^. GC was carried out with a temperature-programmed injection as follows: 50°C for 2 min, then raised by 40°C min^-1^ to 200°C, held for 2 min at 200°C, raised by 3°C min^-1^ to 320°C, and finally held for 30 min at 320°C. Alkanes were determined by GC-MS with the same conditions as above, except for the oven temperature program, which was as follows: 50°C for 2 min, increased by 10°C min^-1^ to 220°C, then raised by 2°C min^-1^ to 260°C, and finally held for 50 min. Both the ion source and the injection port were held at 250°C. The quantitative analysis was carried out using a capillary GC with a flame ionization detector (GC-2014, Shimadzu) under the same conditions as the GC-MS analysis. To determine the fatty acid composition of TAG, fatty acid methyl esters were prepared as described above and analyzed using GC-FID with the following oven temperature program: 180°C for 15 min, and then increased by 2°C min^-1^ to 220°C.

### Phylogenetic Analysis of Surface Lipid Biosynthesis–Related Proteins in *K. flaccidum*

The predicted protein sequences of *K. flaccidum* are available at http://www.plantmorphogenesis.bio.titech.ac.jp/~algae_genome_project/klebsormidium/index.html. Proteins of nine algae, *Chondrus crispus, Ectocarpus siliculosus, Phaeodactylum tricounutum, Cyanidioschyzon merolae, Micromonas* strain RCC299, *Ostreococcus tauri, Chlorella variabilis* NC64A, *Volvox carteri* f. *nagariensis*, and *C. reinhardtii*, and of five land plants, *Physcomitrella patens* subsp. *patens, Selaginella moellendorffii, O. sativa* subsp. *japonica, Populus trichodarpa*, and *A. thaliana*, were compared with those of *K. flaccidum* according to Hori et al. (2014). Based on the protein sequences involved in the biosynthesis of waxes, neutral lipids and cutin monomers in *A. thaliana*, searches using the BLASTP algorithm were carried out using the datasets from these 14 species (e-value < 10^-3^). After removing inadequate sequences that were too short, of low quality, or had large deletions from the phylogenetic analysis, sequences were aligned using the MUSCLE program (Edgar, 2004). Alignments were edited using the G-Blocks program (Talavera and Castresana, 2007) to select the most-conserved sites, and to eliminate both highly variable and/or ambiguous portions of the alignments. The maximum likelihood analyses of all of the datasets were performed with MEGA6.0 (Tamura et al., 2013) using the appropriate models (Le and Gascuel, 2008). Bootstrap analyses with 500 replicates were carried out to estimate the support for internal nodes. These sequences were also studied with InterPro for predicting domains and important sites.

### Cutin Monomer Analysis

Aliphatic components were derived from algal cells or plant according to Bonaventure et al. (2004) with slight modification (Li-Beisson et al., 2013). Approximately 100 mg of algal cells or plant was submerged in hot 2-propanol at 80°C for 10 min. After cooling to room temperature, the tissue was ground with a mortar and pestle and vortexed in the 2-propanol for 1 h to allow complete extraction of whole lipids. After centrifugation at 1,500 *g* for 5 min, the supernatant was discarded and fresh 2-propanol was added to the pellet. The pellet was then re-extracted for 1 h. After centrifugation at 1,500 *g* for 5 min, the supernatant was discarded. The pellet was delipidated sequentially by chloroform/methanol (2:1, v/v; 1 h), chloroform/methanol (1:2, v/v; 1 h), methanol (1 h) for all species. For further delipidation of algae, water (1 h), 2 M NaCl (1 h), water (1 h), methanol (1 h), chloroform/methanol (1:2, v/v; 1 h), chloroform/methanol (2:1, v/v; 1 h) and methanol (1 h) was used as described above. After the delipidation, the solvent was removed under a gentle stream of nitrogen gas, and pellets were lyophilized for 24 h.

To prepare aliphatic monomers, 2 ml of reaction medium, consisting of methanol/methyl acetate/28% sodium methoxide (12:3:5, v/v/v), was added to the delipidated pellets and heated at 60°C for 2 h. After cooling to room temperature, 4 ml dichloromethane, 0.5 ml glacial acetic acid, and 1 ml of buffer [100 mM Tris-HCl (pH 8.0), 0.9% (w/v) NaCl] were added. Phases were separated by centrifugation at 1,500 *g* for 2 min. The lower organic phase was collected, washed with 2 ml of buffer, and dehydrated over anhydrous sodium sulfate. The supernatant was dried under a gentle stream of nitrogen gas. Then, 20 μl of anhydrous pyridine and 20 μl of BSTFA-TMCS were added, and the mixture was vortexed for 10 s and then heated at 70°C for 60 min. After the derivatization, solvents were removed under a gentle stream of nitrogen gas, and lipids were analyzed by GC-MS and GC-FID.

### ATR-FTIR Analysis

Cell wall was extracted as previously described with some modification (Carpita et al., 2001; Moller et al., 2007; Pattathil et al., 2012). Shortly, the pellets lyophilized after delipidation were pre-treated with the following agents: 100 mM 1,2-cyclohexylenedinitrilotetraacetic acid (CDTA) for 1 h at room temperature to remove pectic polysaccharides, 0.34 M NaClO_2_ in 65 mM acetic acid for 1 h at 65°C, 0.1 M NaOH for 1 h at room temperature, or hot water for 1 h at 80°C. Treated pellets were washed with water and then lyophilized for 24 h.

IR spectra were recorded by a Spectrum Two (PerkinElmer Japan Co. Ltd.) with universal attenuated total reflection (UATR) using a single reflection accessory fitted with a diamond prism (incident angle 45°). Dehumidification of inside FTIR system was kept using desiccant (molecular sieve). Pieces of each pellet were mounted on the top of ATR crystal and pressed using pressure arm with check which has 3 mm diameter. For background measurements, the same ATR prism was used without any solution (air only). Spectral measurements were carried out from 4,000 and 450 cm^-1^ at a resolution of 4 cm^-1^ and 24 ± 1°C. Interferograms from 4 scans were averaged to obtain each spectrum. A power spectrum was calculated from the interferogram (both side) using apodization of a Norton-Beer. Obtained original spectra were treated with an ATR correction.

## Results

### SEM Observations

**FIGURE 2:**
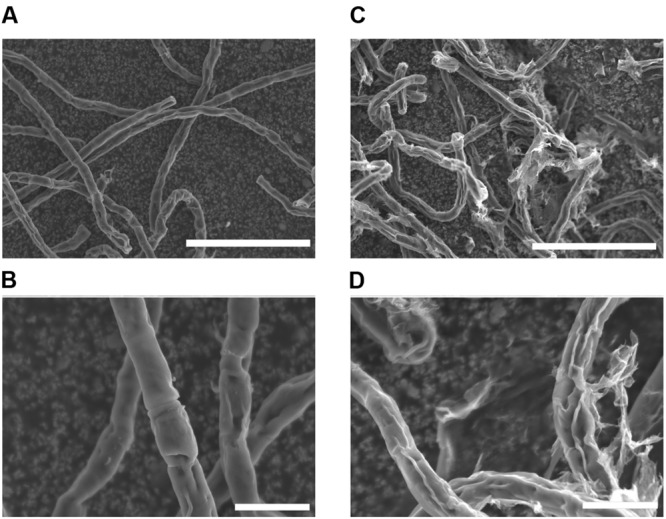
**Scanning electron microscopy pictures of *K. flaccidum* 3 days after transfer to a liquid or solid medium.** SEM pictures of *K. flaccidum* 3 days after transfer to liquid **(A,B)** or solid **(C,D)** medium. Scale bars **(A,C)** = 50 μm, **(B,D)** = 10 μm.

Before investigating surface lipids and their synthesis, surface structure of *K. flaccidum* cells was observed with SEM, 3 days after being transferred to fresh liquid or solid medium. Algal cells transferred to liquid medium had smooth surfaces (**Figures 2A,B**), whereas the surfaces of algae transferred to solid medium were strikingly different and seemed to be coated with frequently disrupted film-like structures (**Figures 2C,D**).

### Phylogenetic Analyses of Surface Lipid Biosynthesis–Related Proteins in *K. flaccidum* and *C. reinhardtii*

We searched protein sequences from *K. flaccidum* and *C. reinhardtii* genomes by BLASTP program on the basis of the protein sequences involved in wax and cutin monomers biosynthesis pathways of *A. thaliana*. These sequences were aligned and curated by ClustalW, MUSCLE and G-Blocks.

### Wax Biosynthesis

**FIGURE 3:**
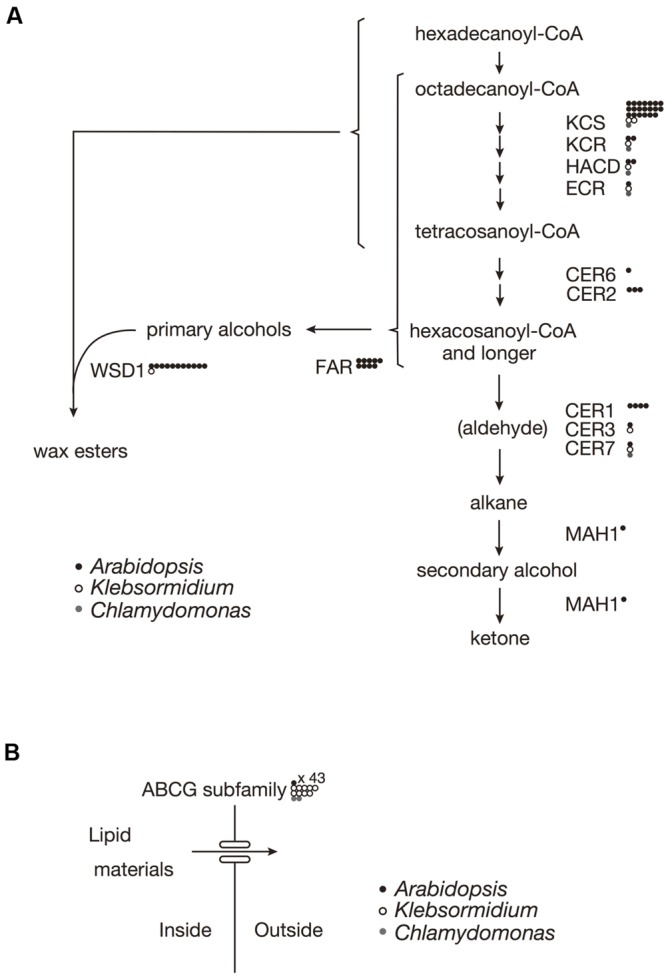
**Gene comparisons among *K. flaccidum, C. reinhardtii* and *A. thaliana*. (A)** Genes involved in the wax biosynthetic pathway. **(B)** Genes involved in the ABCG transporter. Gene homologs for the respective species are shown by the presence of the open and filled circles as indicated. Multiple homologs in a species are represented by multiple circles. Abbreviations: ABCG, ATP binding cassette subfamily G; CER, Eceriferum; ECR, Enoyl-CoA reuctase; FAR, Fatty acid reductase; HACD, Hydroxyacyl-CoA dehydrase; KCR, Ketoacyl-CoA reductase; KCS, ketoacyl-CoA synthase; MAH1, Mid-chain alkane hydroxylase1; WSD, Wax ester synhtase.

In *A. thaliana*, the biosynthesis of almost all of the surface waxes begins with very-long-chain fatty acids (VLCFAs) that typically have ≥C20. Based on a BLAST analysis across genomes, *K. flaccidum* has the counterparts to a set of enzymes involve in the elongation of fatty acids up to C24 in *A. thaliana*. Nevertheless, it is not clear whether *K. flaccidum* is able to synthesize VLCFAs since *A. thaliana* KCS4 could not rescue the yeast *Δelo2Δelo3* double mutant (Paul et al., 2006). *K. flaccidum* does not appear to contain homologs of ECERIFERUM2 (CER2) or ECERIFERUM6 (CER6), which are required for the synthesis of fatty acids containing ≥C28 (**Figure 3A**). CUTI/CER6/KCS6 catalyze the synthesis of VLCFAs with longer aliphatic chains than C24, which are cuticular wax components or precursors (Millar et al., 1999; Todd et al., 1999). CER2 and CER2-like genes are involved in the elongation of C28 to C34 VLCFAs (Haslam et al., 2012, 2015; Pascal et al., 2013). For alkane biosynthesis, there are counterparts to both ECERIFERUM3 (CER3) and ECERIFERUM7 (CER7) in *K. flaccidum* that work indirectly in the decarbonylation pathway. For *C. reinhardtii*, homologs involving fatty acid elongation, namely ketoacyl-CoA synthase (KCS), ketoacyl-CoA reductase (KCR), hydroxyacyl-CoA dehydrogenase (HACD), and enoyl-CoA reductase (ECR), were identified. No counterpart to ECERIFERUM1 (CER1), which produces alkanes, was clearly identified. Midchain alkane hydroxylase1 (MAH1) is involved in two steps in *A. thaliana*, the oxidization of alkane to a secondary alcohol, followed by the oxidization of the secondary alcohol to a ketone (Greer et al., 2007). *K. flaccidum* does not appear to have a MAH1 homolog. *A. thaliana* fatty acid reductase (FAR) protein sequences, when queried against the *K. flaccidum* genome using the BLASTP algorithm, produced no significant hits. However, only one gene encoding wax ester synthase/acyl-coenzyme A: diacylglycerol acyltransferase1-like (WSD1-like) was found in *K. flaccidum*. In case of *C. reinhardtii*, only a CER7 homolog was found in alkane biosynthesis, and no counterpart to WSD1-like proteins or FAR was identified in wax ester biosynthesis.

### ABCG Transporters

In *A. thaliana*, some ABCG transporters are responsible for the transportation of wax precursors (Bird et al., 2007; Panikashvili et al., 2007; Panikashvili and Aharoni, 2008). *K. flaccidum* appears to contain homologs of ABCG transporters (**Figure 3B**). These ABCG transporters work as dimers, for example, DESPERADO (DSO)/ABCG11 and CER5/ABCG12 form heterodimers for transporting the wax precursors to the epicuticular wax layer. In the *K. flaccidum* and *C. reinhardtii* genomes, only counterparts to DSO were found.

### Qualitative Analysis of Extracts by Immersion in Chloroform

**FIGURE 4:**
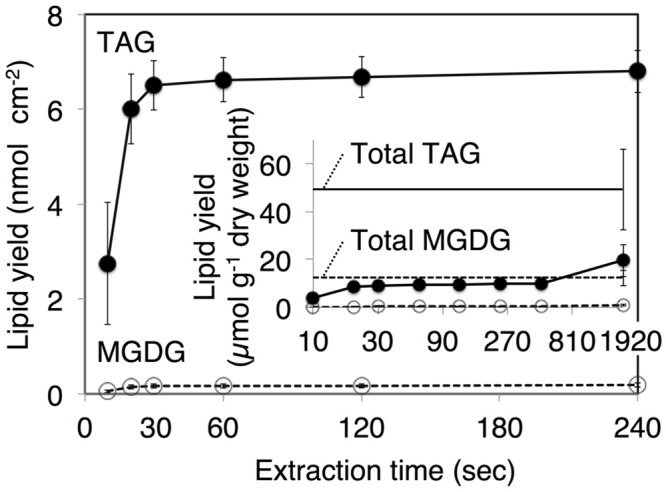
***Klebsormidium flaccidum* lipid recovery yields according to the different extraction time points.** The *K. flaccidum* lipids were extracted by chloroform. Solvent was collected at predefined times and fresh solvent was added immediately. Closed circles with solid lines indicate TAG, whereas open circles with dotted lines indicate MGDG. The inset shows lipid recovery per dry weight, and the solid and dotted straight lines represent TAG and MGDG, respectively, which were both derived using the Bligh and Dyer method (Bligh and Dyer, 1959). Values represent means ± SD (*n* = 3).

**FIGURE 5:**
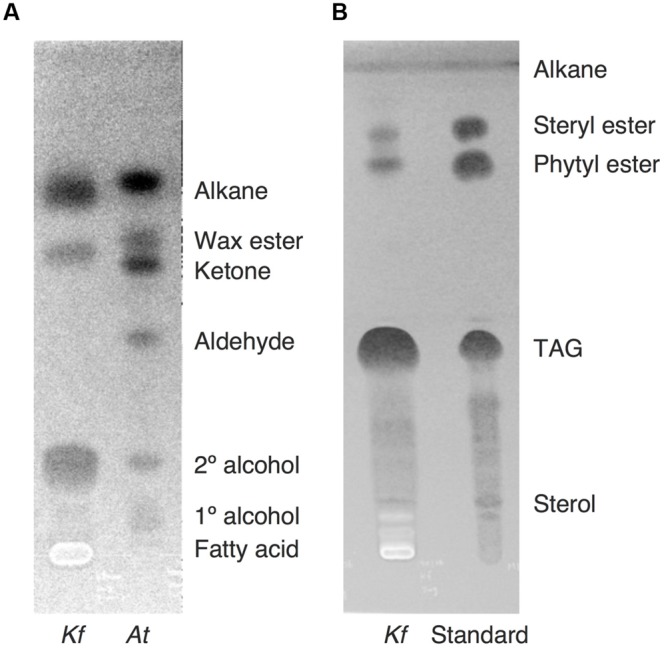
**Lipid separation extracted from *K. flaccidum* and *A. thaliana* using the chloroform extraction method. (A)** Comparison of lipid separation between *K. flaccidum* and *A. thaliana*. The lipids of *K. flaccidum* were extracted once with chloroform for 30 s, whereas those of *A. thaliana* were extracted twice with chloroform and the resulting lipids were pooled. Lipids were separated on a TLC plate using a solvent system containing hexane/diethyl ether/acetic acid (90:7.5:1, v/v/v), and visualized with 0.01% (w/v) primuline in 80% (v/v) acetone under ultraviolet light. **(B)** Lipid separation of *K. flaccidum*. Lipids extracted with chloroform as in **(A)** were separated on a TLC plate using the following solvent system: hexane to a height of 20 cm and, toluene to a height of 20 cm, followed by hexane/diethyl ether/acetic acid (70:30:1, v/v/v) to a height of 10 cm.

**FIGURE 6:**
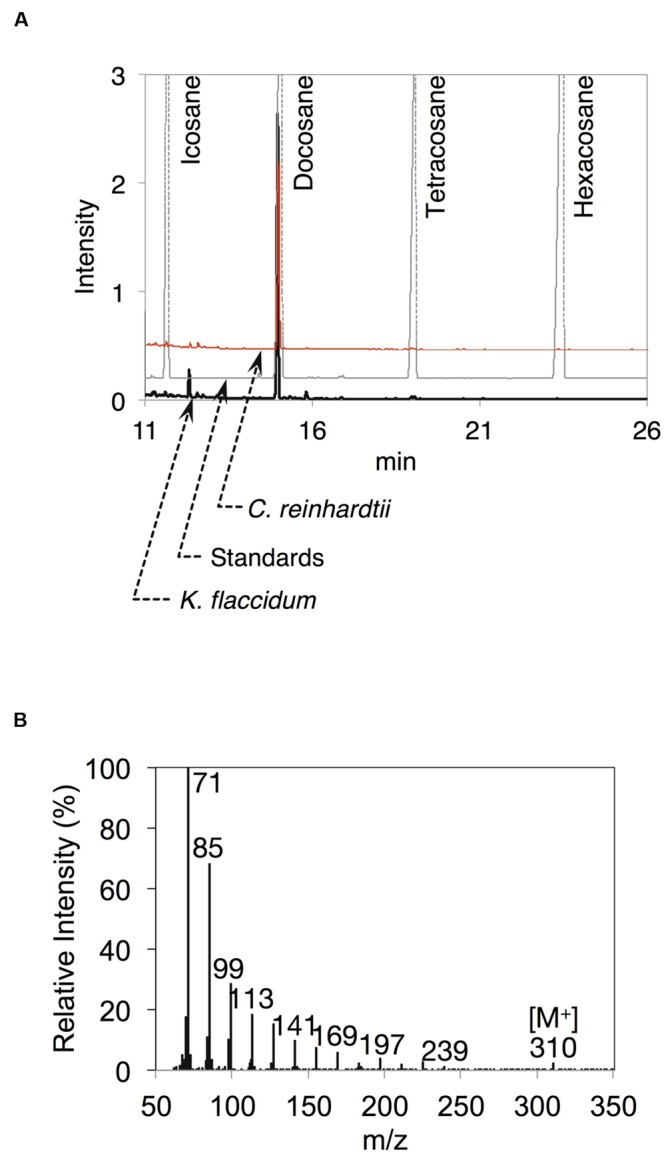
**Alkanes extracted from *K. flaccidum* and *C. reinhardtii* using the chloroform extraction method. (A)** GC-FID chromatogram of the alkane fraction of *K. flaccidum* extracted with chloroform. Lipids were compared with commercially available standard alkanes. Black line: *K. flaccidum*. Red line: *C. reinhardtii.* Gray line: standards. **(B)** EI-MS spectrum of docosane obtained from *K. flaccidum*.

After the identification of several genes related to surface lipid biosynthesis in *K. flaccidum* genome, we used two different methods to extract surface lipids from both *K. flaccidum* and *C. reinhardtii*. First, we applied the chloroform submergence method for extracting wax from plants (Li et al., 2008). In the initial assessment, immersion time points were studied using *K. flaccidum*. From 10 to 30 s, the lipid recovery rose sharply and then gradually increased until 1,920 s (**Figure 4**). Thus, algal lipids were extracted with chloroform for 30 s. We note that the lipid yield at 1,920 s contained ∼40% of the total amount of TAG and 7.6% of the total amount of MGDG obtained by the Bligh and Dyer method (**Figure 4**). After extraction, lipids were separated on a TLC plate, identified by GC-MS, and quantified by GC-FID. According to the TLC images, the lipid composition of *K. flaccidum* was different from that of *A. thaliana* (**Figure 5A**). The fractions were identified as alkanes, steryl esters, phytyl esters, TAGs, and sterols; however, ketones, aldehydes, and primary and secondary alcohols were not detected (**Figures 5A,B**). To determine their compositions, lipids were extracted from spots and analyzed by GC-MS after hydrolysis with sodium methoxide and/or derivatization to trimethylsilyl ethers if necessary. Both *K. flaccidum* and *C. reinhardtii* included only docosane in their alkane fractions in which odd-numbered alkanes were absent according to the GC-FID chromatograms (**Figure 6A**) and GC-MS spectra (**Figure 6B**). Steryl esters, which included phytosterols, campesterol, β-sitosterol, and stigmasterol as the steryl moiety, were found only in *K. flaccidum* (Supplementary Figures S1A–D and S2A). Although *K. flaccidum* had an alkyl ester, its alcohol moiety consisted of phytol instead of fatty alcohol. The composition of the phytyl ester was very simple, as only a single ester, phytyl palmitate, was found in the GC-FID chromatogram (Supplementary Figure S1E). GC-MS spectra corroborated that the ester contained a phytyl moiety (Supplementary Figure S1F). The abundant fatty acids in the TAG were linoleic acid (18:2) and oleic acid (18:1) in *K. flaccidum* and *C. reinhardtii*, respectively (Supplementary Figure S2C). The fatty acid composition of TAG corresponded with that of the inner TAG in *K. flaccidum* (Supplementary Figure S2C), whereas the composition was similar to that of the inner TAG in *C. reinhardtii* grown under nitrogen starvation conditions (Iwai et al., 2014). The final members of the algal surface lipids were the sterols, which reflected the composition of steryl esters in *K. flaccidum* (Supplementary Figures S1G and S2B).

### Qualitative Analysis of Surface Lipids Obtained by Exposure to Silica Gel Plates

**FIGURE 7:**
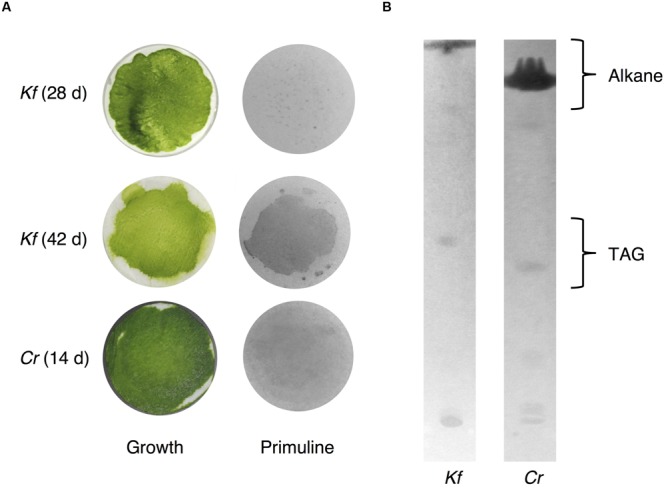
**Wax extracted from *A. flaccidum* using the silica gel extraction method. (A)** Left, top to bottom: 28 days old and 42 days old *K. flaccidum* cultures, and 14 days old *C. reinhardtii*. Right: lipid recovery from silica gel plate extractions for *K. flaccidum* and *C. reinhardtii*. **(B)** Separation of lipid from *K. flaccidum* and *C. reinhardtii*. Lipids derived by silica gel plate extraction were separated on a TLC using the following solvent system: hexane to a height of 20 cm and, toluene to a height of 20 cm, followed by hexane/diethyl ether/acetic acid (70:30:1, v/v/v) to a height of 10 cm.

Because we cannot exclude the possibility that exposure to chloroform, even for a short period of time, could result in the extraction of intracellular lipids from *K. flaccidum* and *C. reinhardtii*, we tried to establish an alternative extraction method for these microorganisms by briefly placing them on a silica gel plate. We also applied this method, with slight modification, including contact with gentle pressure, to the acquisition of wax from *A. thaliana* (Supplementary Figure S3). Wax separation on a TLC plate indicated that the silica gel extraction method could be applied to *A. thaliana* since seven neutral lipids were extracted to the same extent as chloroform extraction (Supplementary Figure S3B). To determine the appropriate contact time, *K. flaccidum* (cultured for 42 days) grown on a nitrocellulose membrane was laid culture side down on a TLC plate for predetermined times from 30 s to 20 min. MGDG was not detected even after 20 min. Supplementary Figure S4 shows that 5 min was necessary and sufficient for the lipid extraction and, therefore, 5 min was used as the contact time for lipid extraction by silica gel plates. We also determined the appropriate culture time for *K. flaccidum* for the lipid extraction. Pale patches, indicative of lipid fluorescence, were observed in the primuline image of *K. flaccidum* cultured for 28 days (**Figure 7A**), but, by comparison, a darker and broader fluorescence was found from algae cultured for 42 days (**Figure 7A**). Therefore, algae cultured for 42 days were used for the lipid analyses. The same extraction method was applied to *C. reinhardtii* cultured for 14 days. Although the fluorescence intensity was weaker than that of *K. flaccidum* cultured for 42 days, lipids were also obtained from *C. reinhardtii* using this method (**Figure 7A**). In both algae, the lipids obtained by the silica gel plate method consisted of similar classes as those from the chloroform extracts. More specifically, lipids from the silica gel plate extractions included alkanes and TAGs; however, steryl esters, phytyl esters, and sterols were not found (**Figure 7B**). All of the constituents of the lipids acquired by silica gel extraction were included in those acquired by chloroform, and no additional lipid classes were detected.

### Comparison of the Lipid Contents Isolated with the Two Extraction Methods

**FIGURE 8:**
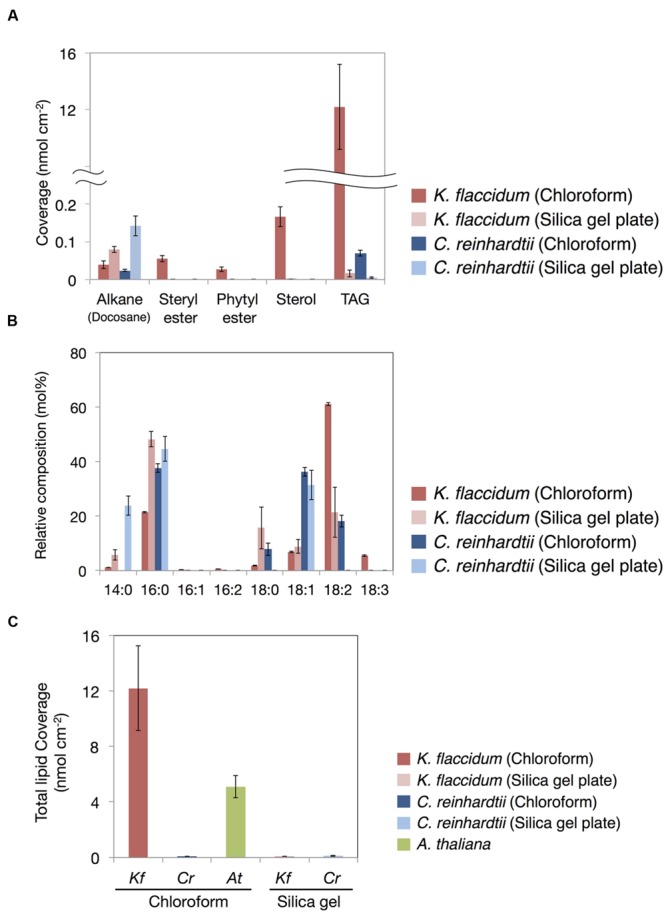
**Waxes from *K. flaccidum, C. reinhardtii*, and *A. thaliana*. (A)** Comparison of the composition of waxes extracted from *K. flaccidum* and *C. reinhardtii* using different extraction methods. Waxes were separated by TLC, and appropriately derivatized and characterized by GC-FID. **(B)** Comparison of the composition of TAG fatty acids extracted from *K. flaccidum* and *C. reinhardtii* using different extraction methods. **(C)** Comparison of the total content of waxes from *K. flaccidum, C. reinhardtii*, and *A. thaliana* using different extraction methods. Values represent means ± SD (*n* = 4).

**Figure 8A** shows the lipid composition of each lipid class of algae and plant extracted using the chloroform and silica gel methods. The docosane content for both *K. flaccidum* and *C. reinhardtii* was higher when using the silica gel plate extraction method compared to the chloroform extraction.

The fatty acid content of the TAG fraction from *K. flaccidum* seemed to differ depending on the extraction method. **Figure 8B** shows that hexadecanoic acid (16:0) was the major component extracted by the silica gel plate method, whereas octadecadienoic acid (18:2) was the most abundant component in the chloroform extract. Furthermore, the molar ratios of tetradecanoic acid (14:0) and octadecanoic acid (18:0) in the silica gel plate extraction were higher than those in the chloroform extraction. The same trend was observed in *C. reinhardtii.* In the silica gel plate extraction of *C. reinhardtii*, the molar ratios of tetradecanoic acid and hexadecanoic acid were higher than those in the chloroform extraction, and only octadecenoic acid (18:1) was detected in C18 fatty acids.

The amounts of surface lipids in the three organisms obtained by different extraction methods are shown in **Figure 8C**. The lipid content extracted from *K. flaccidum* with chloroform was 12.2 nmol cm^-2^, which was ∼120 times higher than the amount obtained by silica gel plate extraction. In contrast, the amount of lipids obtained from *C. reinhardtii* by chloroform extraction was slightly less, at 0.09 nmol cm^-2^, than that obtained by silica gel plate extraction, at 0.13 nmol cm^-2^. The wax load derived from *A. thaliana* was 5.1 nmol cm^-2^, which was the highest value among all of the surface lipids, except for the wax load of *K. flaccidum* obtained by chloroform extraction.

### Analysis of Aliphatic Cell Wall Components in Algae

**FIGURE 9:**
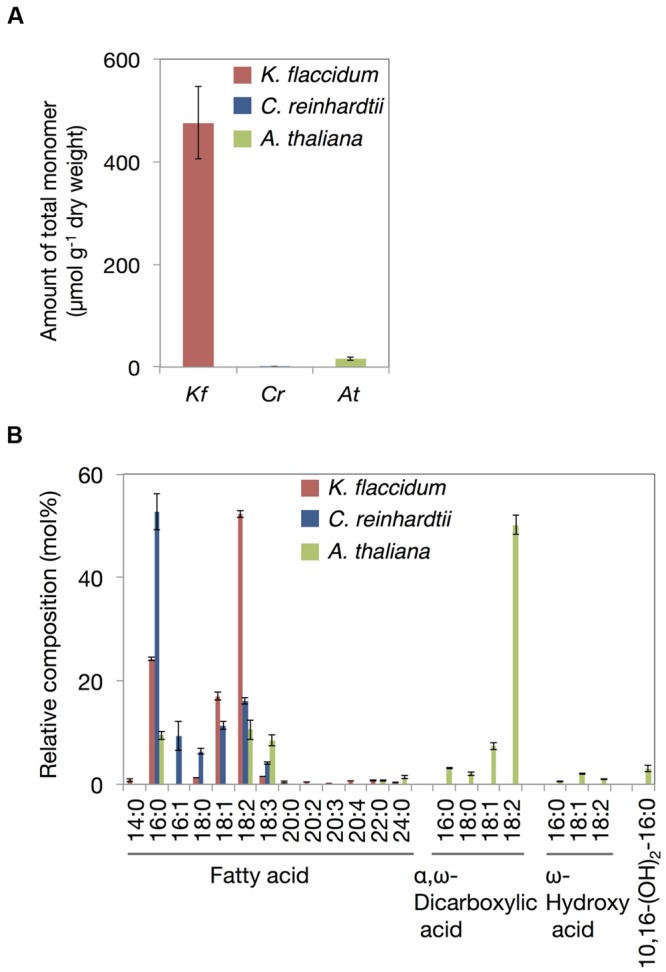
**Cutin-like monomers from *K. flaccidum, C. reinhardtii*, and *A. thaliana* cell wall residue. (A)** Comparison of total cutin-like monomers between algae and plants. **(B)** Comparison of cutin-like monomers in algae and plants. Values represent means ± SD (*n* = 4).

Based on the method for analyzing cutin in *A. thaliana*, aliphatic components included in the delipidated algal residues were analyzed after alkaline hydrolysis followed by the appropriate derivatization. **Figure 9A** shows the total monomer contents of *K. flaccidum, C. reinhardtii*, and *A. thaliana*. Surprisingly, *K. flaccidum* had a considerable amount of fatty acid, ∼30-fold higher than that of *A. thaliana*. The amount of aliphatic monomers in *C. reinhardtii* was quite low compared with those of *K. flaccidum* and *A. thaliana*. **Figure 9B** shows the composition of the aliphatic components in the three organisms. Contrary to *A. thaliana*, which contains ω-hydroxy acid, 10,16-dihydroxy palmitic acid, and α,ω-dicarboxylic acid, key components of the cuticular network in higher plants (Yeats and Rose, 2013), *K. flaccidum* and *C. reinhardtii* contain only simple linear carbon chain fatty acids as aliphatic constituents that attach to cell wall components. No other fatty acid derivatives, like those in *A. thaliana*, were found in *K. flaccidum* or *C. reinhardtii*. In *K. flaccidum*, octadecadienoic acid (18:2) was the most abundant monomer, followed by hexadecanoic acid (16:0) and octadecenoic acid (18:1), and the contents of other fatty acids were very low. In *C. reinhardtii*, hexadecanoic acid was the most abundant component, followed, in decreasing order, by octadecadienoic acid (18:2), octadecenoic acid (18:1), hexadecenoic acid (16:1), and octadecanoic acid (18:0).

### ATR-FTIR Analyses of Delipidated Cell Wall Residues

**FIGURE 10:**
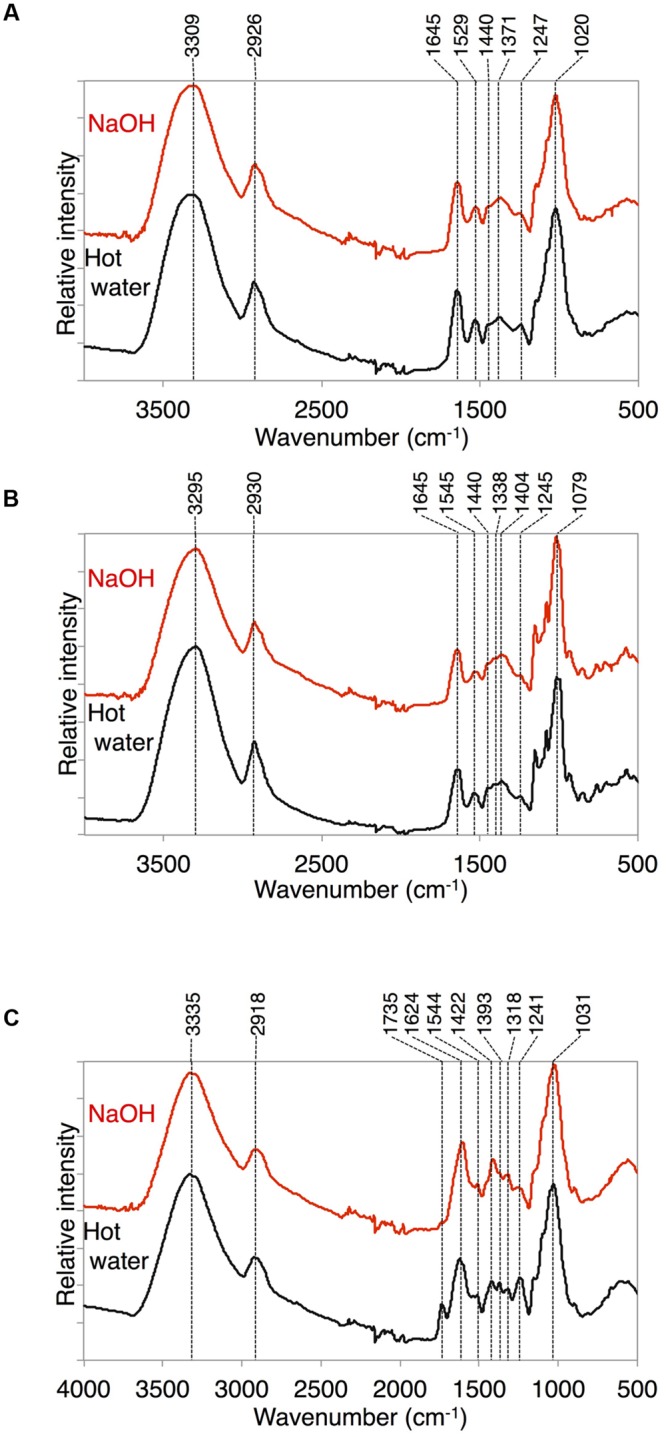
**ATR-FTIR spectra obtained from *K. flaccidum, C. reinhardtii*, and *A. thaliana*.** ATR-FTIR spectra obtained from *K. flaccidum* **(A)**, *C. reinhardtii* **(B)**, and *A. thaliana* **(C)** cell wall residues after NaOH and hot water extractions.

Because we found a large amount of fatty acid monomers that were covalently attached to the delipidated cell wall fraction in *K. flaccidum*, we analyzed the ATR-FTIR spectra of these fractions to determine how the fatty acids were attached to the cell wall. As shown in **Figure 10C** for *A. thaliana*, a peak at 1735 cm^-1^ was observed after the hot water treatment and then lost after the 0.1 M NaOH treatment. This peak probably represented C = O stretching and, therefore, the ester bond originating from the cuticle layer may have been largely removed by the NaOH treatment in *A. thaliana*. However, the corresponding peaks were originally much weaker in *K. flaccidum* and *C. reinhardtii*, and no change was observed after the same treatments (**Figures 10A,B**). Instead, two amide peaks at 1645 and 1529–1545 cm^-1^ were obvious, indicating that the represented proteins are major components of the cell wall fractions.

## Discussion

Only a few studies on the extracellular lipids of microorganisms have been reported. Conidiospores of *Alternaria tenuis, Botrytis fabae*, and *Neurospora crassa*, and sporangiospores of *Rhizopus stolonifer*, were shown to have extracellular lipids (Fisher et al., 1978). In that study, the immersion of fungal conidiospores and sporangiospores in an organic solvent was used to extract lipids from the spore surface, but it was not clear whether lipids were derived only from the surface. To further understand the extracellular lipids of microorganisms, we tried to develop a new methodology for the extraction of outer lipids from algae using a silica gel plate instead of an organic solvent. Our approach was based on a previous study that used silica gel particles in the extraction of lipids from insects (Choe et al., 2012). For algae, it is difficult to separate silica gel particles from algal cells because of the fineness of the particles. Hence, we used a silica gel plate for lipid extraction from *K. flaccidum* and *C. reinhardtii*. Lipid extraction using a silica gel plate appeared to be useful in analyzing surface lipids of microalgae in comparison to the chloroform extraction method. However, all of the extracellular lipids might not have been recovered by this method. The compositions of the lipid classes were different between the extraction methods. We speculate that the differences between these methods reflect how lipids exist on the surface of the cells. Alkanes, sterols, steryl esters, phytyl esters, and TAGs were isolated by chloroform extraction, whereas only alkanes and TAGs were detected following silica gel plate extraction from *K. flaccidum*. The amount of alkanes found was similar to that of steryl esters and phytyl esters and was lower than that of sterols by chloroform extraction. The alkane yield was higher in the silica gel plate extraction than in the chloroform extraction in both *K. flaccidum* and *C. reinhardtii*. Silica gel plate extraction probably favors alkanes over other lipids, resulting in the higher alkane yield compared with other lipid yields.

Comprehensive genomic homology searches suggested that *K. flaccidum* and *C. reinhardtii* do not produce VLCFAs of more than 28 carbons (**Figure 3A**). Likewise, very-long-chain fatty alcohols might not be formed in these organisms because of the absence of fatty acid reductase genes. Thus, we speculated that wax esters with long-chain fatty acids and fatty alcohols are not present in *K. flaccidum* in spite of the presence of a WSD1-like counterpart. This was supported by our subsequent surface lipid analysis, which indicated that these plant-like wax components were not found and that the surface lipid components were much more primitive, being mainly composed of alkanes and TAGs. These components, particularly a large proportion of TAGs, are probably embedded in the primitive cuticle layer, which is composed of cell wall–attached single fatty acyl chains.

*Arabidopsis thaliana* surface lipids contain alkanes composed of odd-numbered chains, such as nonacosane and hentriacontane (Supplementary Figure S5), whereas *K. flaccidum* and *C. reinhardtii* surface lipids contain an even-numbered chain alkane, docosane (**Figures 6A,B**). The presence of even-numbered chain alkanes is not unusual, as they are found in several organisms, including the yeast *Saccharomyces oviformis*, the fungus *Trichoderma viride* (Ladygina et al., 2006), and the bacterium *Vibrio furnissii* M1 (Park, 2005). In *A. thaliana*, odd-numbered chain alkanes are synthesized through the decarbonylation pathway from VLCFAs (Kunst and Samuels, 2009). In contrast, even-numbered chain alkanes are proposed to be synthesized by the reduction of primary alcohols via aldehydes in *V. furnissii* (Park, 2005). Although *K. flaccidum* appears to contain counterparts to *A. thaliana* CER3 and CER7, which are involved in alkane biosynthesis, CER1, another important factor in this pathway, has not been found. CER3 and CER7 work indirectly and CER1 is necessary for alkane synthesis (Bourdenx et al., 2011; Bernard et al., 2012). It is not clear whether CER3 and CER7 are involved in the formation of docosane. The assumed proteins involved in phytyl esters and steryl esters syntheses in *K. flaccidum* were shown in Supplementary Figures S6 and S7, respectively.

### Comparison of the TAG Composition between Extraction Methods

It is interesting that the fatty acid composition of TAGs recovered by silica gel plate extraction was distinct from that recovered by chloroform extraction (**Figure 8B**). The former was composed of more saturated fatty acids than the latter from both *K. flaccidum* and *C. reinhardtii*. This result could be expected, as unsaturated fatty acids are auto-oxidized more readily than saturated fatty acids under aerobic conditions (Porter et al., 1995). Indeed, many terrestrial plants have waxes composed of saturated fatty acid derivatives. Furthermore, the differences in the fatty acid constituents from intracellular TAGs are likely to demonstrate the existence of extracellular TAG in addition to the intracellular TAG fraction. Supplementary Figure S8 shows a proposed pathway for TAG biosynthesis in *K. flaccidum* and the candidate genes involved. Even though the *K. flaccidum* genome has a low number of gene paralogs in general (Hori et al., 2014), when compared with *A. thaliana, K. flaccidum* contains the minimum set of enzymes required to assemble TAG. Moreover, *K. flaccidum* might carry a counterpart to diacylglycerol acyltransferase 1 (DGAT1), which catalyzes the final step of TAG formation and is proposed to play a role in sequestering the fatty acids of galactolipids released during chloroplast senescence (Kaup et al., 2002). Hence, a similar mechanism for salvaging fatty acids from galactolipids may occur in *K. flaccidum* and *C. reinhardtii* during some stress responses.

Recently, it was reported that Bayberry fruit contained a unique surface wax composed of major amounts of DAG and TAG, and minor amounts of MAG, all of which included only saturated fatty acids (Simpson and Ohlrogge, 2016). In their report, TAG was thought to be synthesized from DAG as an acyl acceptor and MAG as a donor outside the cell via unknown pathway. Three cutin-associated acyltransferases, *sn*-2 GPATs (Li et al., 2007a,b; Yang et al., 2010), an HXXXD-motif acyltransferase closely related to *Arabidopsis* DEFECTIVE IN CUTICULAR RIDGES (DCR) (Panikashvili et al., 2009) and GDSL-motif lipase/transacylases related to tomato CUTIN DEFICIENT 1 (CD1) (Yeats et al., 2012), seem to involve in the TAG synthesis in Bayberry. Although *K. flaccidum* does not appear to have the counterparts to *sn*-2 GPAT or DCR, the counterparts to close homolog of CD1 from *A. thaliana*, Cutin synthase-like protein(LTL1/AtCUS1) (Yeats et al., 2014) and GDSL-MOTIF LIPASE5 (GLIP5) (Oh et al., 2005), were found (Supplementary Table S1). Other counterparts to HXXXD-motif acyltransferases analyzed biologically in *A. thaliana* were not identified in *K. flaccidum*. It is possible that the counterparts to LTL1/AtCUS1 or GLIP5 might involve in the surface TAG synthesis in *K. flaccidum* in a similar fashion to that in Bayberry fruit, however, the pathway is still unclear since our data are insufficient because of the lack of their biochemical analyses.

### Aliphatic Monomers Derived from the Cell Wall Fraction

When *K. flaccidum* was transferred to a solid medium, film-like structures were observed on the surface (**Figures 2C,D**); these structures were absent when the alga was cultured in liquid medium (**Figures 2A,B**). The film-like structures may have resulted from the drier growth conditions. Barberousse et al. (2006) described structures in *K. flaccidum* that were made from polysaccharides absorbed in higher amounts on hydrophobic than on hydrophilic materials. This alga may enwrap itself in more aliphatic substances than only polysaccharides, and these films may contain secreted surface lipid components that prevent water loss. In fact, appreciable amounts of fatty acids were detected in the *K. flaccidum* cell wall residue (**Figures 9A,B** and Supplementary Figure S9). Although *C. reinhardtii* includes hydroxyproline-rich glycoproteins on the cell wall (Goodenough and Heuser, 1985), it also has small amounts of fatty acids in its cell wall residue.

In both *K. flaccidum* and *C. reinhardtii*, the FTIR spectra for hot water, chlorite, and CDTA treatments were similar to each other (Supplementary Figure S10), which reflects the absence of a plant-like hemicellulose-like polymers in *K. flaccidum*, as indicated in a previous study (Sørensen et al., 2011). The differences in FTIR spectra (**Figure 10C**) between hot water and 0.1 N NaOH treatments in *A. thaliana* indicated that the peak at 1735 cm^-1^ corresponded to ester bonds binding to the wall matrix. Given that *K. flaccidum* has few ester bonds, similar to *C. reinhardtii*, according to its FTIR spectrum (**Figures 10A,B**), a large proportion of fatty acids is likely to bind to cell wall components, such as the lysine residues of cell wall proteins, via amide bonds. Indeed, two amide peaks, at 1645 and 1529–1545 cm^-1^, which probably correspond to amide I and amide II, respectively, were found in both *K. flaccidum* and *C. reinhardtii*. Because the cell walls of *C. reinhardtii* seem to lack cellulose, and are largely composed of extracellular glycoproteins (Roberts, 1974), these amide bonds in *K. flaccidum* may be due to cell wall glycoproteins. Very small peaks corresponding to ester bonds in algae demonstrated that there was little contamination by TAGs or other esters in these fractions. Thus, the aliphatic moieties in the *K. flaccidum* cell wall seem to be in the form of single fatty acyl chains, and, therefore, most TAGs, sterols, steryl esters, and phytyl esters may be embedded in the cell wall through their hydrophobic interactions with the fatty acyl moieties of the cell wall. The level of TAG obtained by chloroform extraction was ∼730-fold higher than that obtained by silica gel plate extraction in *K. flaccidum*. However, the level of TAG obtained by chloroform extraction was only 14-fold higher than that obtained by silica gel plate extraction in *C. reinhardtii*. Thus, the amount of TAG embedded in the cell wall of *C. reinhardtii* is probably much less than that of *K. flaccidum.*

Weak, pale fluorescence was observed from *K. flaccidum* cultured for 28 days, whereas a stronger fluorescence was observed at 42 days, which indicated that the amount of lipids excreted to the surface increased with aging (**Figure 7**). Three of the five lipids detected in the chloroform extraction from *K. flaccidum* were esters (**Figure 8A**), which also play a role in the detoxification of fatty acid and alcohol (Turkish et al., 2005; Bouvier-Navé et al., 2010; Lippold et al., 2012). Thus, extracellular lipids are likely to be secreted as waste products and might function as defensive substances on the surface and within the acylated cell walls of *K. flaccidum*.

**FIGURE 11:**
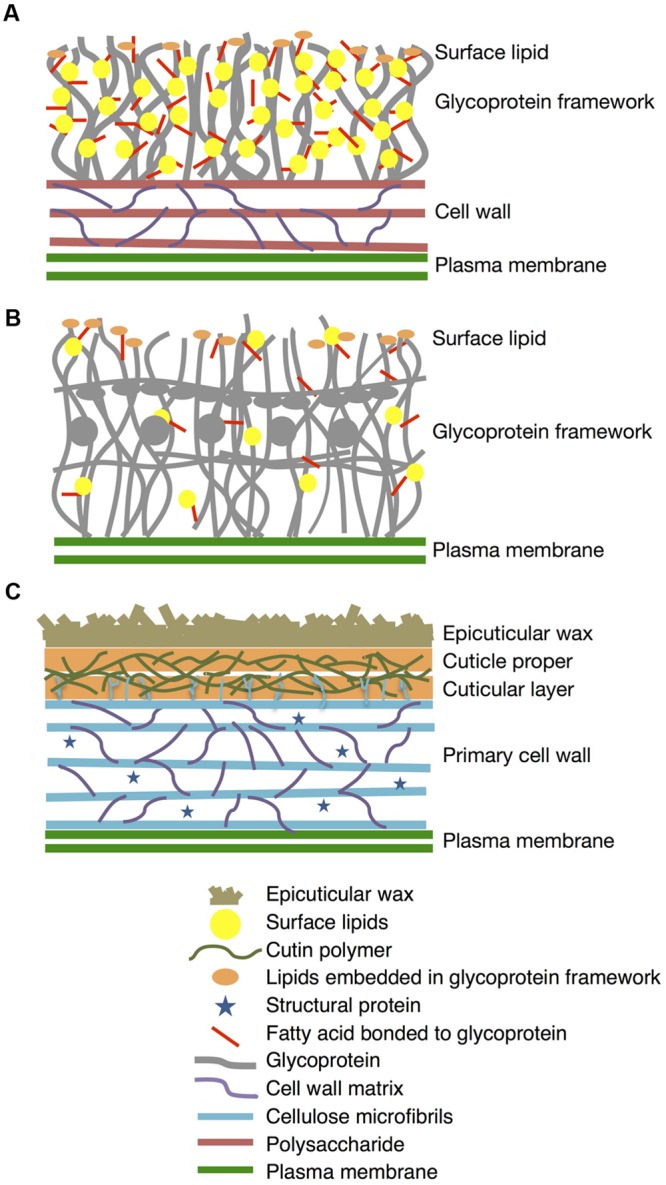
**Diagrams of lipophilic layers on cell walls of *K. flaccidum, C. reinhardtii*, and *A. thaliana*.** Proposed diagrams for lipophilic layers and their connection to the cell walls of *K. flaccidum* **(A)**, *C. reinhardtii* **(B)** and *A. thaliana* **(C)**. Spatial distribution, orientation, size and population of all constituents in this diagram are not per scale.

In this work, we present for the first time that *K. flaccidum* has lipophilic layers distinct from those of *A. thaliana* that contain a rather primitive wax layer and an alternative lipid-protein complex rather than a cutin polymer (**Figure 11**). *K. flaccidum* most likely has a glycoprotein framework with acyl side chains on its cell wall, with some lipids embedded in this framework. *C. reinhardtii* might also possess glycoproteins with acyl side chains, but the quantities of side chains and lipids in the framework are relatively small. Although the system to protect against stress conditions is more primitive than that in land plants, these aliphatic layers likely enable the algae to survive under terrestrial conditions.

## Author Contributions

SK performed GC-FID, GC-MS, and computational analysis. KH prepared samples of *K. flaccidum* strain NIES-2285 *C. reinhardtii*, and *A. thaliana*. KH helped in computational analysis. YS-S helped in GC-MS analysis. AK performed SEM analysis. TK and NY-O performed ATR-FTIR analysis. TN helped in lipid preparation. KO helped in cultivation of *K. flaccidum*. SK and HO wrote the manuscript and all authors contributed to the manuscript revision. MS and HO planned the project.

## Conflict of Interest Statement

The authors declare that the research was conducted in the absence of any commercial or financial relationships that could be construed as a potential conflict of interest.

The reviewer SR and handling Editor declared their shared affiliation, and the handling Editor states that the process nevertheless met the standards of a fair and objective review.
