# Supplementary material for: Primitive Extracellular Lipid Components on the Surface of the Charophytic Alga Klebsormidium flaccidum and Their Possible Biosynthetic Pathways as Deduced from the Genome Sequence
Source: Front Plant Sci. 2016 Jun 30;7:952. doi: 10.3389/fpls.2016.00952 (PMC4927632; doi:10.3389/fpls.2016.00952)
Supplement: Supplementary file 1 [file Data_Sheet_1.PDF]

Supplementary materials

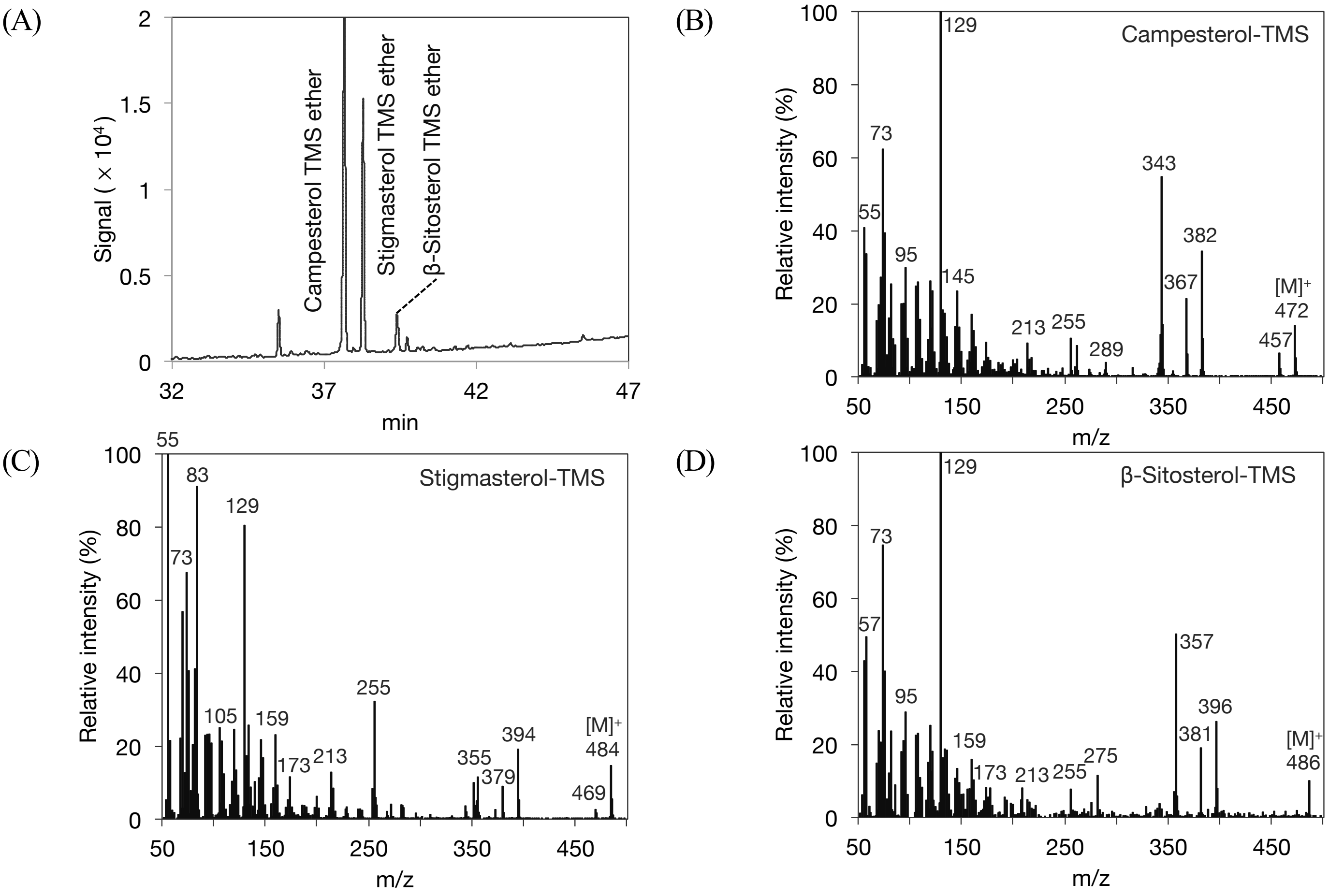

Supplementary Figure 1 |

GC-FID chromatogram and EI-MS spectra of sterol moiety of steryl ester, non-hydrolyzed phytyl ester and free sterol extracted from 28-d-old *K. flaccidum* using the chloroform extraction method.

(A) GC-FID chromatogram of the sterol moiety of steryl ester. The steryl ester was measured after alkaline hydrolysis with 2 M sodium methoxide.

(B) EI-MS spectrum of campesterol trimethylsilyl (TMS) ether.

(C) EI-MS spectrum of stigmasterol TMS ether.

(D) EI-MS spectrum of β-sitosterol TMS ether.

(E)

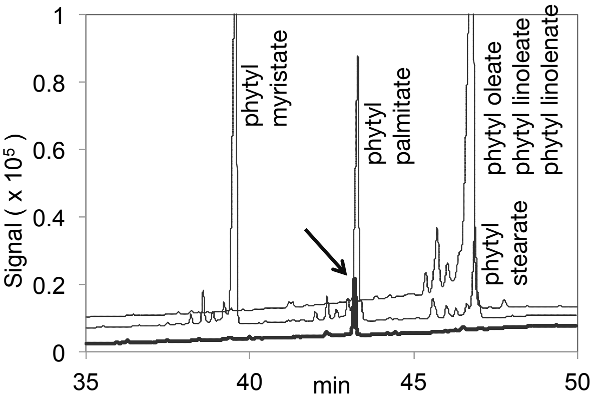

(F)

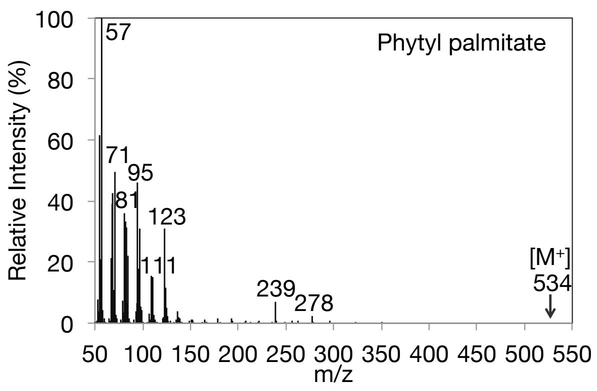

(G)

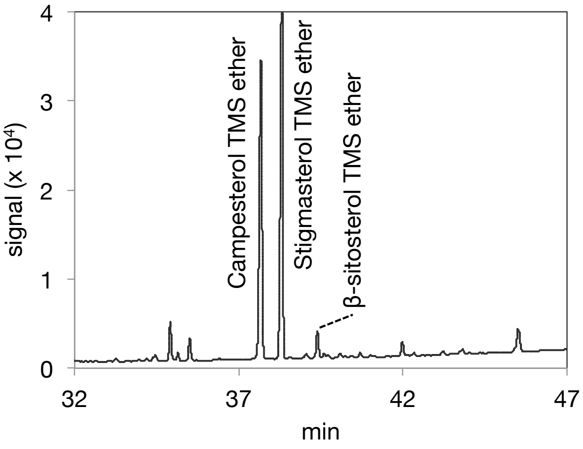

Supplementary Figure 1 (continued)|

(E) GC-FID chromatogram of non-hydrolized phytyl ester. Extracted lipids (black line) were compared with synthesized phytyl esters (gray line).

(F) EI-MS spectrum of phytyl palmitate.

(G) GC-FID chromatogram of free sterol.

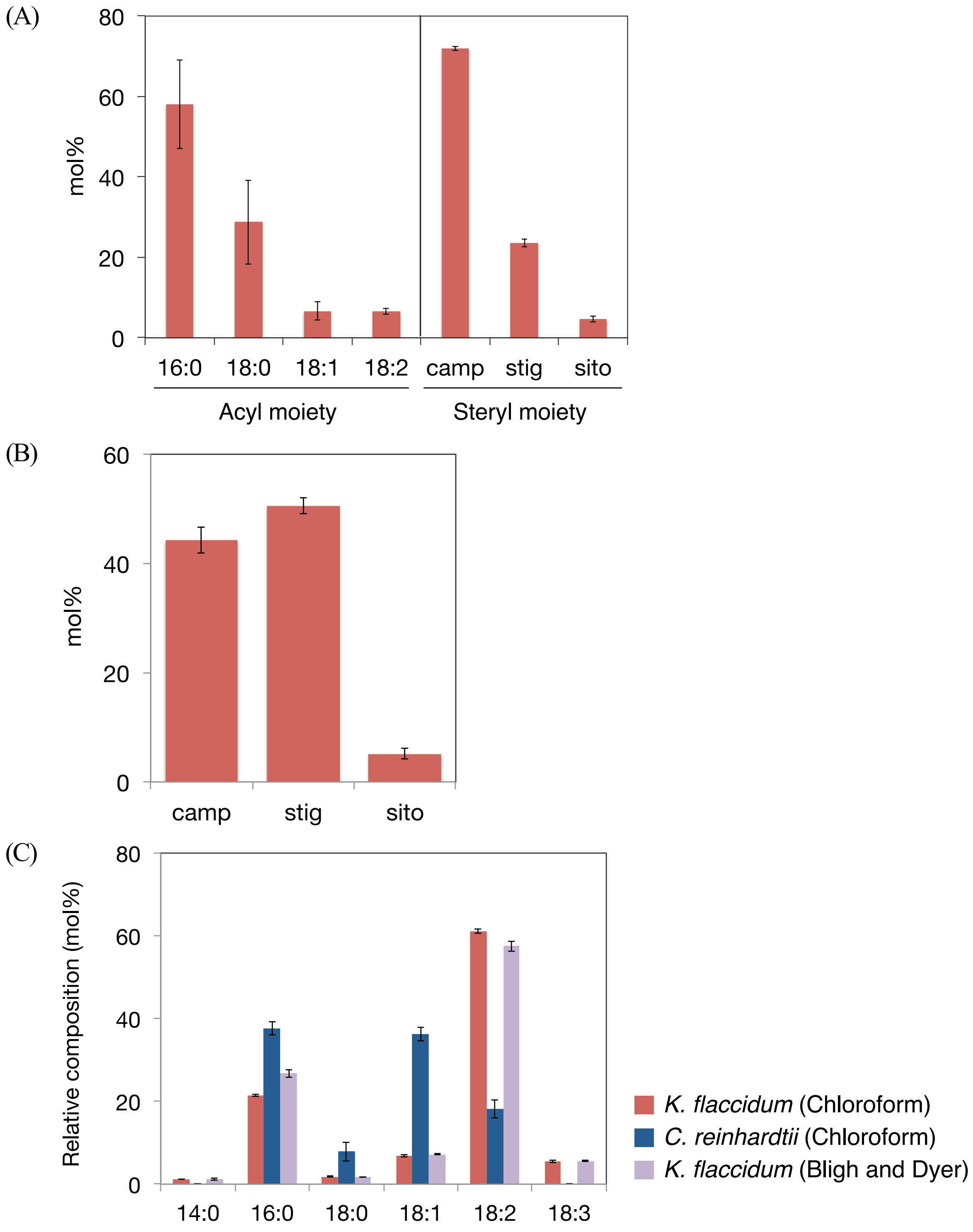

Supplementary Figure 2 |

Relative composition of acyl and sterol moieties of steryl ester and free sterols extracted from 28-d-old *K. flaccidum* using the chloroform extraction method.

(A) Composition of acyl and sterol moieties. Abbreviation: camp, campesterol; stig, stigmasterol; sito, β-sitosterol.

(B) Composition of free sterols. Abbreviation: camp, campesterol; stig, stigmasterol; sito, β-sitosterol.

(C) Fatty acid composition of surface TAG extracted with chloroform from *K. flaccidum* and *C. reinhardtii*, and inner TAG extracted from *K. flaccidum* using the method by Bligh and Dyer. The values of surface TAG extracted with chloroform from both algae were the same as those in Figure 8(B). Values represent means ± SD (n = 4 for surface TAG and n=3 for inner TAG).

(A)

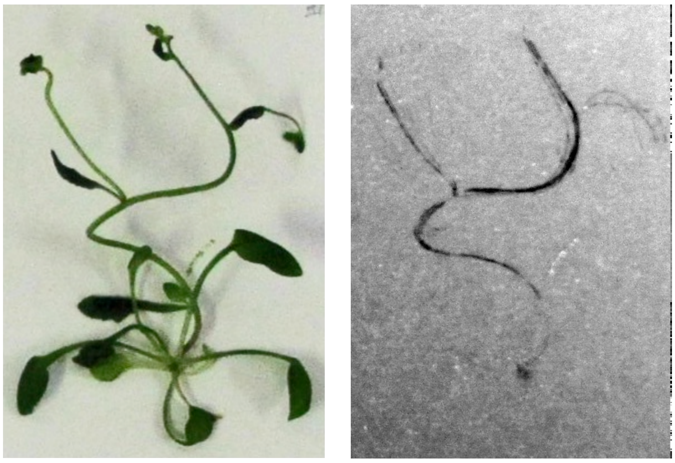

(B)

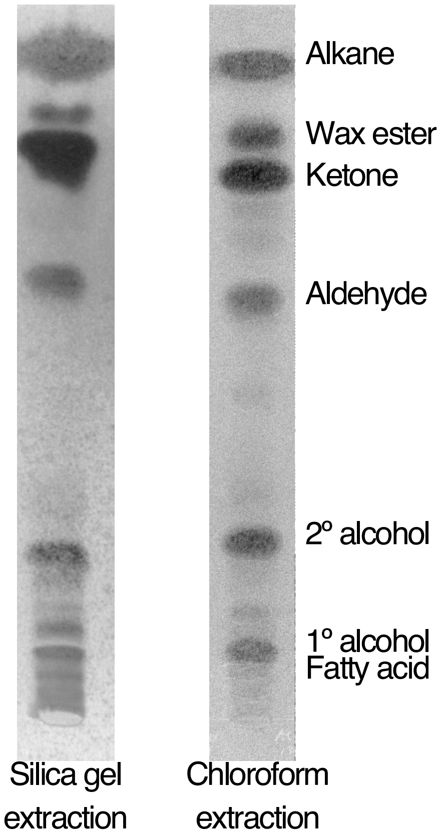

Supplementary Figure 3 |

Comparison of wax recoveries between different extraction methods in 31-d-old *A. thaliana.*

(A) Wax of *A. thaliana* extracted by a silica gel plate. Left image represents the growth image, and right image represents primuline fluorescence image. Stems of *A. thaliana* were depressed gently on a silica gel plate three times without scraping. The pressing force was ~300 g cm^{-2}.

(B) Comparison of waxes extracted from *A. thaliana* using different extraction methods. Left image represents wax separation extracted with silica gel plate, and right image represents that with chloroform. Waxes were separated on a TLC plate using the solvent system containing hexane/diethyl ether/acetic acid (90:7.5:1, v/v/v).

(A)

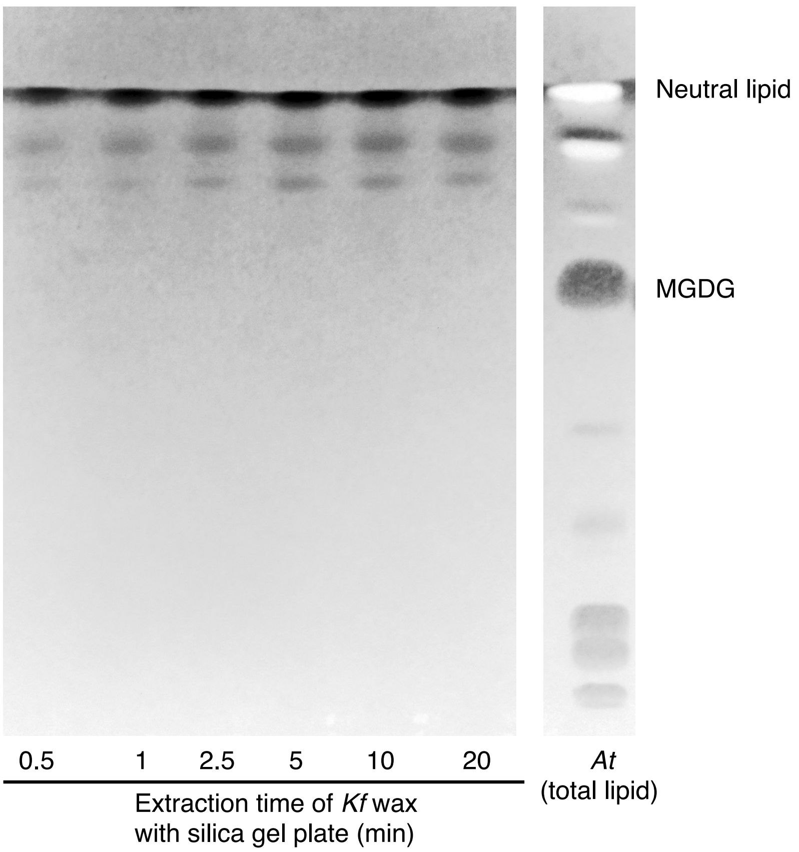

(B)

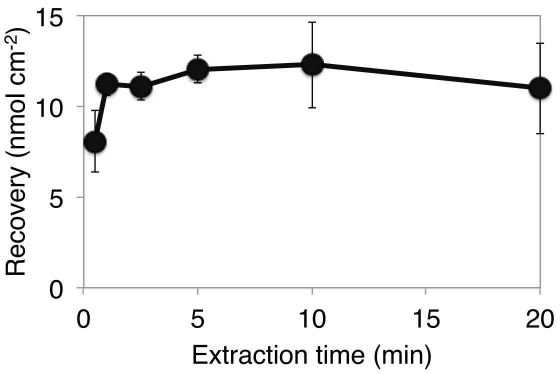

Supplementary Figure 4

Determination of extraction time from 42-d-old *K. flaccidum* using silica gel plate.

(A) *K. flaccidum* grown on a nitrocellulose membrane was placed growing surface down on a silica gel 60 TLC plate for predefined periods. Lipids were recovered and separated on a TLC plate by using the following sequential two-part development system: first, to a height of 20 cm with hexane/diethyl ether/acetic acid (70:30:1, v/v/v), and, second, to a height of 10 cm with acetone/toluene/water (91:30:8, v/v/v).

(B) Neutral lipids in (A) were scanned and quantified by ImageJ software with co-migrated standard. Values represent mean ± SD (n = 2).

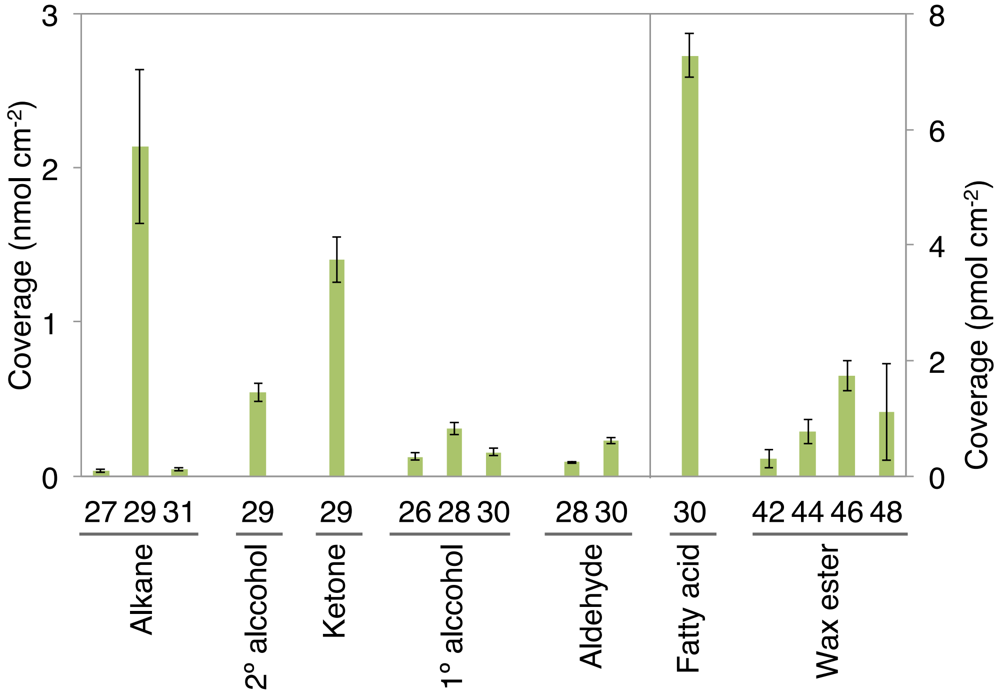

Supplementary Figure 5 |

Composition of waxes extracted from 42-d-old *A. thaliana.* Stems of *A. thaliana* were submerged twice in chloroform for 30 sec each time, and waxes were recovered from the chloroform. Waxes were concentrated and separated with TLC and then characterized by GC-MS and GC-FID. Values represent means ± SD (n = 4).

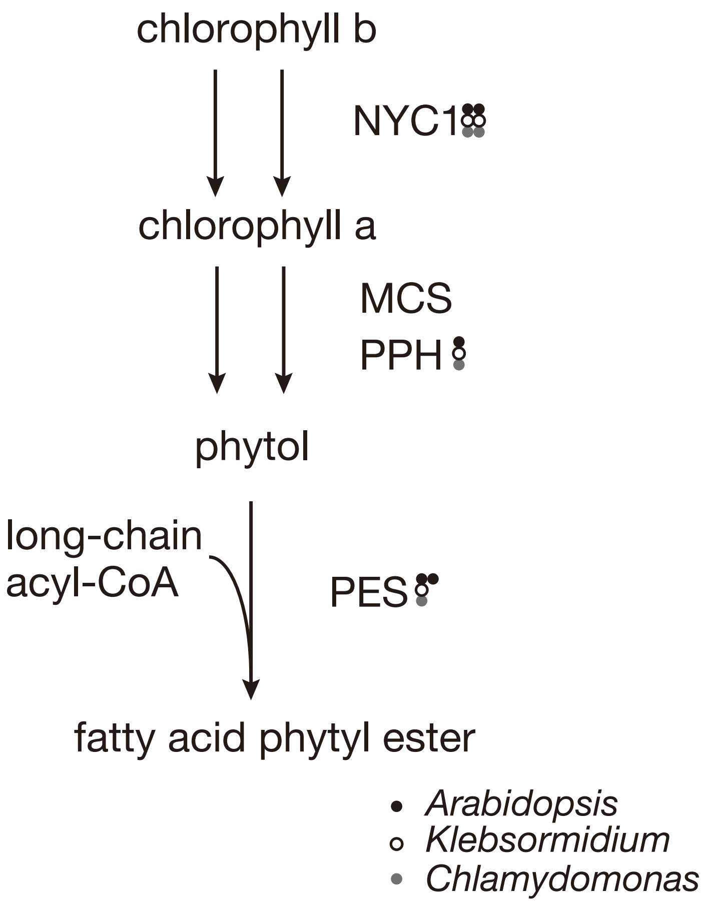

Supplementary Figure 6 |

Comparison of genes involved in the phytyl ester biosynthetic pathways of *K. flaccidum*, *C. reinhardtii* and *A. thaliana*. Gene homologs for the respective species are shown by the presence of the open and filled circles as indicated. Multiple homologs in a species are represented by multiple circles. Abbreviations: MCS, Metal-chelating substance; NYC1, Non-yellow coloring1; PES, Phytyl ester synthase; PPH, Pheophytinase.

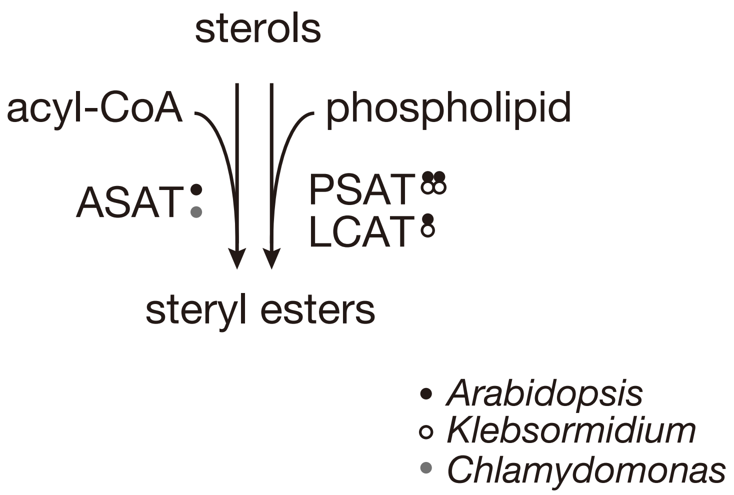

Supplementary Figure 7 |

Comparison of genes involved in the steryl ester biosynthetic pathways of *K. flaccidum*, *C. reinhardtii* and *A. thaliana*. Gene homologs for the respective species are shown by the presence of the open and filled circles as indicated. Multiple homologs in a species are represented by multiple circles. Abbreviations: ASAT, Acyl-CoA sterol acyltransferase; LCAT, Lecithin: cholesterol acyltransferase; PSAT, Phospholipid sterol acyltransferase.

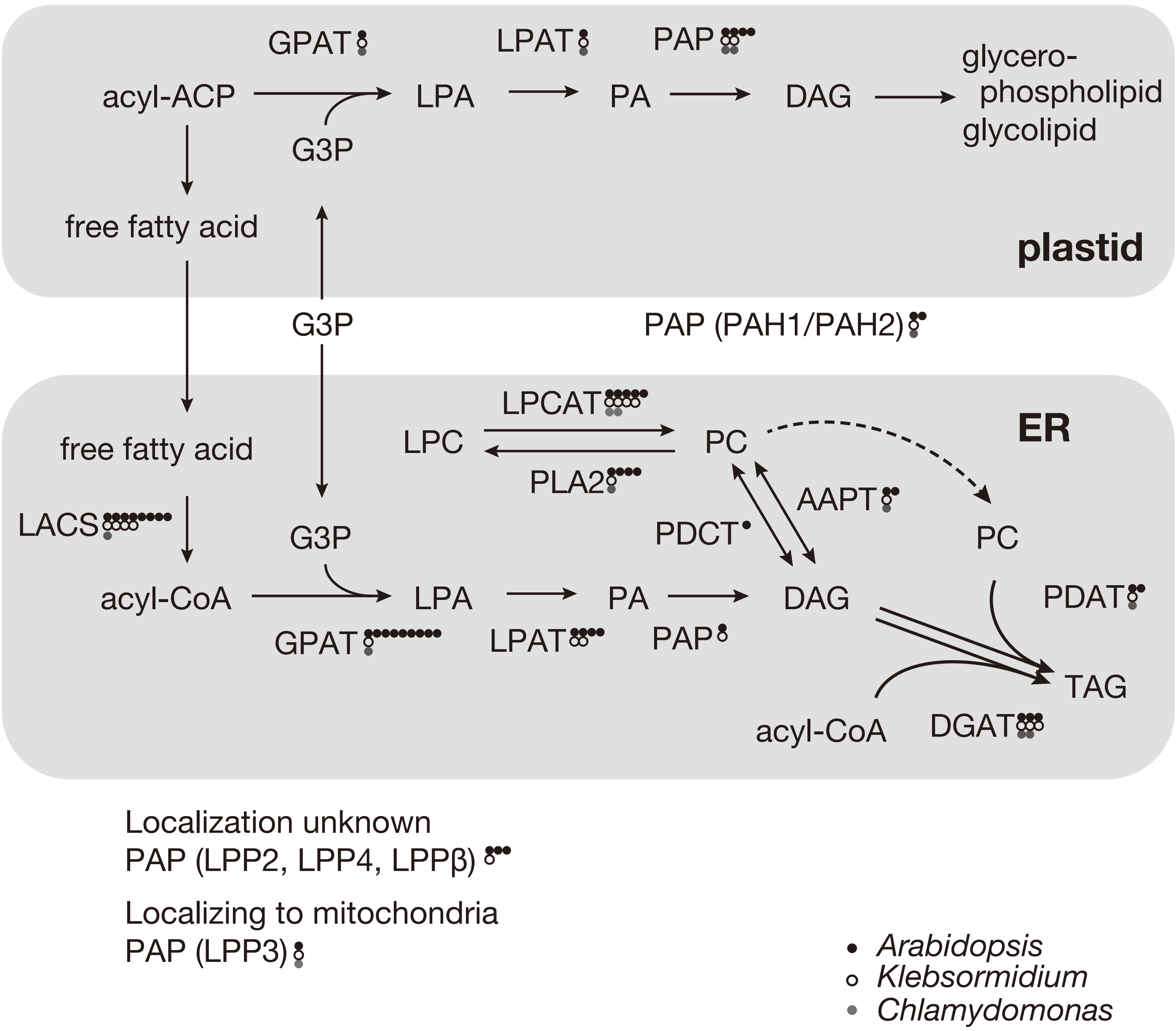

Supplementary Figure 8 |

Comparison of genes involved in the TAG biosynthetic pathways of *K. flaccidum*, *C. reinhardtii* and *A. thaliana*.
Gene homologs for the respective species are shown by the presence of the open and filled circles as indicated. Multiple homologs in a species are represented by multiple circles. Abbreviations: ER, Endoplasmic reticulum; AAPT, Aminoalcoholphosphotransferase; ACP, Acyl Carrier Protein; DGAT, Diacylglycerol acyltransferase; GPAT, Glycerol-3-phosphate *sn*-2-acyltransferase; LACS, Long-chain acyl-CoA synthetase; LPAT, Lysophosphatidyl acyltransferase; LPCAT, Lysophosphatidylcholine:acyl-CoA acyltransferase; PAP, Phosphatidate phosphatase; PDAT, Phospholipid:diacylglycerol acyltransferase; PDCT, Phosphatidylcholine diacylglycerol cholinephosphotransferase; PLA2, Phospholipase A2: DAG, Diacylglycerol; G3P, Glycerol-3-Phosphate; LPA, Lysophosphatidic acid; PA, Phosphatidic acid; PC, Phosphatidylcholine.

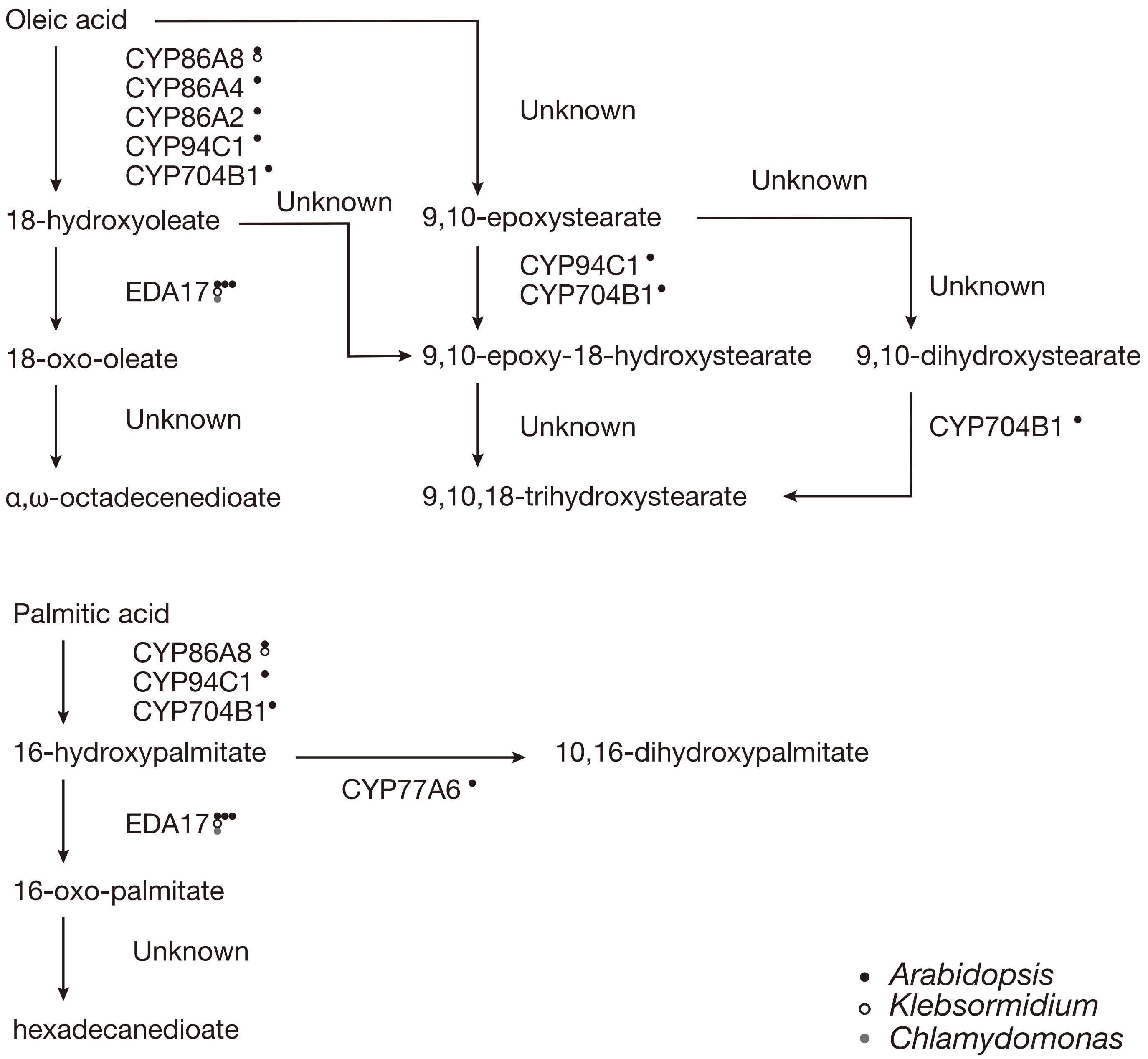

Supplementary Figure 9 |

Comparison of genes involved in the cutin monomer biosynthetic pathways of *K. flaccidum*, *C. reinhardtii* and *A. thaliana*. Gene homologs for the respective species are shown by the presence of the open and filled circles as indicated. Multiple homologs in a species are represented by multiple circles. Abbreviations: CYP, Cytochrome P450; EDA17, Embryo sac development arrest 17.

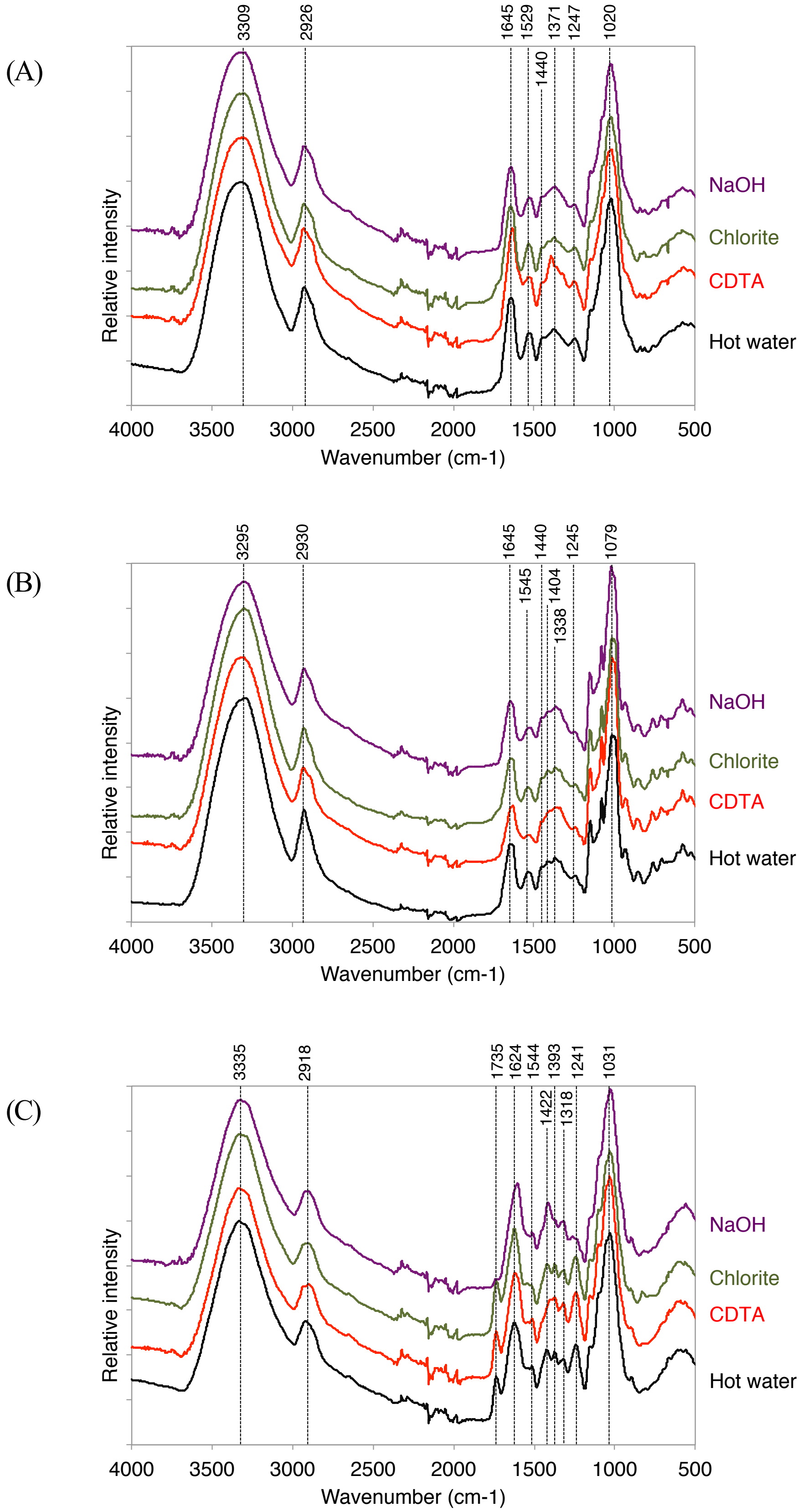

Supplementary Figure 10 |

ATR-FTIR spectra obtained from *A. thaliana*, *K. flaccidum*, and *C. reinhardtii*.

(A) ATR-FTIR spectra of 42-d-old *K. flaccidum.* From the top, NaOH, chlorite, cyclohexane diamine tetraacetic acid (CDTA) and hot water treatments.

(B) ATR-FTIR spectra of 14-d-old *C. reinhardtii* under the same conditions as in (A).

(C) ATR-FTIR spectra of 42-d-old *A. thaliana* under the same conditions as in (A).

Supplementary Table 1 |

Assignment of candidate genes encoding enzymes involved in lipid metabolism in *K. flaccidum*.

Pathway	Description	Locus	Abbreviation	Candidate Counterpart in *K. flaccidum*	e-value	Reciprocal e-value	Candidate Counterpart in *C. reinhardtii*	e-value	Reciprocal e-value
TAG biosynthesis	sn1-Glycerol-3-phosphate acyltransferase	AT1G32200	ATS1/ACT1	kfl00319_0110	3.0E-153	5.0E-154	Cre02.g143000.t1.2	1.0E-99	1.0E-99
		AT5G60620	GPAT9	kfl00139_0130	4.0E-173	1.0E-172	g6130.t1	3.0E-132	2.0E-132
	1-Acylglycerol-3-phosphate acyltransferase	AT1G75020	LPAT4	kfl00129_0110	3.0E-107	6.0E-107	No blast hit	-	-
		AT3G57650	LPAT2	kfl00079_0020	2.0E-119	4.0E-119	No blast hit	-	-
		AT4G30580	LPAT1	kfl00653_0060	1.0E-80	2.0E-80	g9888.t1	5.0E-84	6.0E-84
	Phosphatidate phosphatase	AT3G02600	LPP3	kfl00214_0110	9.0E-70	6.0E-70	Cre05.g230900.t1.3	6.0E-36	8.0E-40
		AT4G22550	LPPβ	kfl00352_0080	3.0E-24	8.0E-22	No blast hit	-	-
		AT5G03080	LPPγ	kfl00010_0330	2.0E-56	4.0E-56	Cre06.g272400.t1.2	1.0E-23	1.0E-23
		AT3G58490	LPPδ	kfl00063_0290	6.0E-84	2.0E-91	No blast hit	-	-
		AT5G66450	LPPε2	kfl00001_0550	3.0E-33	9.0E-34	Cre06.g295250.t1.3	1.0E-30	2.0E-32
		AT5G42870	PAH2	kfl00577_0080	1.0E-145	3.0E-145	Cre12.g506600.t1.2	7.0E-68	2.0E-13
	Diacylglycerol cholinephosphotransferase	AT3G25585	AAPT2	kfl00450_0090	5.0E-145	8.0E-145	Cre12.g538450.t1.2	9.0E-36	1.0E-35
	Phosphatidylcholine:diacylglycerol cholinephosphotransferase	AT3G15820	PDCT	No blast hit	-	-	No blast hit	-	-
	1-Acylghycerol-3-phosphocholine acyltransferase	AT1G12640	LPLAT1	kfl00025_0100	8.0E-160	6.0E-151	g13221.t1	5.0E-68	2.0E-69
		AT1G78690	LPLAT	kfl00573_0090	3.0E-71	4.0E-71	No blast hit	-	-
		AT1G80950	LPEAT1	kfl00255_0160	1.0E-98	1.0E-96	Cre17.g70730.t1.2	1.0E-56	7.0E-59
		AT2G45670	LPEAT2	kfl00898_0040	1.0E-104	2.0E-104	No blast hit	-	-
	Oleate desaturase	AT3G12120	FAD2	kfl00096_0060	2.0E-163	4.0E-163	Cre17.g711150.t1.2	1.0E-129	1.0E-129
	Linoleate desaturase	AT2G29980	FAD3	No definite counterpart		-	No definite counterpart		-
	Phospholipase A2	AT2G19690	PLA2β	kfl00026_0180	4.0E-39	2.0E-38	Cre02.g095000.t1.2	3.0E-29	1.0E-29
	Phospholipid:diacylglycerol acyltransferase	AT5G13640	PDAT1	kfl00076_0090	0	0	Cre02.g106400.t1.3	3.0E-56	1.0E-56
	Acyl-CoA:diacylglycerol acyltransferase	AT1G48300	DGAT3	kfl00422_0070	6.0E-07	4.0E-07	No blast hit	-	-
		AT3G51520	DGAT2	kfl00368_0010	4.0E-97	7.0E-97	Cre03.g205050.t1.2	4.0E-56	3.0E-59
		AT2G19450	DGAT1	kfl00874_0010	5.0E-166	2.0E-164	g1030.t1	4.0E-70	1.0E-72
	Monoacylglycerol acyltransferase	AT1G52760	MAGAT	No blast hit	-	-	No blast hit	-	-
	Choline kinase	AT1G74320	CEK2	kfl00254_0100	2.0E-107	1.0E-112	No blast hit	-	-
		AT2G26830	CEK4	kfl00447_0070	6.0E-110	1.0E-109	No blast hit	-	-
	Choline-phosphate cytidylyltransferase	AT2G32260	CCT1	kfl00623_0050	3.0E-120	5.0E-118	No blast hit	-	-
Coat protein	oleosine	AT4G25140	OLE1	No blast hit	-	-	No blast hit	-	-
	steroleosin	AT5G50600	HSD1	kfl00097_0040	4.0E-75	1.0E-74	No blast hit	-	-
	caleosin	AT2G33380	ATCLO3	kfl00014_0110	9.0E-70	2.0E-69	Cre06.g287000.t1.2	9.0E-59	5.0E-59
WAX biosynthesis	Ketoacyl-CoA synthase	AT1G19440	KCS4	kfl00169_0130	0	0	Cre17.g722150.t1.2	0	0
		AT1G19440	KCS4	kfl00132_0240	0	0	No blast hit	-	-
	Ketoacyl-CoA reductase	AT1G67730	KCR1	kfl00058_0310	7.0E-112	1.0E-111	No blast hit	-	-
		AT1G24470	KCR2	No blast hit	-	-	g9724.t1	2.0E-25	2.0E-25

Supplementary Table 1 (continued)|

Assignment of candidate genes encoding enzymes involved in lipid metabolism in *K. flaccidum*.

Pathway	Description	Locus	Abbreviation	Candidate Counterpart in *K. flaccidum*	e-value	Reciprocal e-value	Candidate Counterpart in *C. reinhardtii*	e-value	Reciprocal e-value
WAX biosynthesis	Hydroxyacyl-CoA dehydratase	AT5G10480	PAS2	kfl00054_0250	2.0E-64	3.0E-64	Cre03.g167950.t1.2	3.0E-49	9.0E-51
	Enoyl-CoA reductase	AT3G55360	ECR	kfl00019_0590	1.0E-112	1.0E-124	Cre14.g615050.t1.2	2.0E-104	1.0E-105
	Acyl-CoA thioesterase	AT1G01710	ACT2	kfl00409_0050	3.0E-94	5.0E-105	No blast hit	-	-
	Long-chain acyl-CoA synthetase	AT2G47240	LACS1	kfl00227_0090	0	0	Cre13.g566650.t2.1	0	0
		AT4G23850	LACS4	kfl00274_0140	0	0	No blast hit	-	-
		AT5G27600	LACS7	kfl00607_0030	0	0	No blast hit	-	-
		AT2G04350	LACS8	kfl00027_0510	0	0	No blast hit	-	-
	A component of the fatty acid elongation machinery required for C28 to C30 fatty acid elongation	AT4G24510	CER2	No blast hit	-	-	No blast hit	-	-
	Alcohol forming fatty acid reductase	AT5G22500	FAR1	No blast hit	-	-	No definite counterpart		-
	Putative aldehyde decarbonylase	AT1G02190	CER1	No blast hit	-	-	No blast hit	-	-
		AT5G57800	CER3	kfl00392_0170	8.0E-72	0	No blast hit	-	-
	Positively regulates CER3 transcription, involved in cuticular wax biosynthesis	AT3G60500	CER7	kfl00020_0140	2.0E-119	3.0E-122	Cre03.g175000.t1.2	3.0E-85	6.0E-87
		AT1G57750	CYP96A1/MAH1	No blast hit	-	-	No blast hit	-	-
Ester biosynthesis	A bifunctional enzyme, wax ester synthase (WS) and diacylglycerol acyltransferase (DGAT)	AT3G49210	WSD1-like	kfl00431_0050	2.0E-63	3.0E-64	No blast hit	-	-
	Phospholipid sterol acyl transferase 1	AT1G04010	PSAT1	kfl00160_0010p	0	0	No blast hit	-	-
	Phosphoserine aminotransferase 2	AT2G17630	PSAT2	kfl00085_0160	0	0	No blast hit	-	-
	Acyl-CoA sterol acyl transferase 1	AT3G51970	ASAT1	No blast hit	-	-	Cre07.g349900.t1.3	9.0E-22	3.0E-21
	Lecithin:cholesterol acyltransferase 3	AT4G19860	-	kfl00069_0190	4.0E-126	6.0E-150	No blast hit	-	-
	Phytyl ester synthase	AT1G54570	PES1	kfl00048_0280	3.0E-165	3.0E-174	Cre12.g521650.t1.2	9.0E-110	3.0E-116
Chlorophyll degradation	Chlorophyllase	AT1G19670	CLH1	No blast hit	-	-	Cre03.g148750.t1.2	9.0E-12	9.0E-12
		AT5G43860	CLH2	kfl00505_0030	7.0E-47	6.0E-45	g12031.t1	3.0E-12	9.0E-12
	Chlorophyll b reductase	AT4G13250	NYC1	kfl00003_0160	4.0E-134	9.0E-134	Cre12.g517700.t1.2	2.0E-81	5.0E-84
		AT5G04900	NYC1-like	kfl00376_0070	1.0E-114	3.0E-114	Cre14.g608800.t1.2	2.0E-113	2.0E-113
	Pheophytinase	AT5G13800	PPH	kfl00254_0150	3.0E-99	3.0E-106	Cre12.g514700.t1.2	4.0E-82	5.0E-83
Transporter	ABCG half transportert	AT1G17840	ABCG11	kfl00574_0100	0.0E+00	0.0E+00	Cre02.g096000.t2.1	1.0E-173	3.0E-168
Cutin monomer synthesis	CYP86 clan	AT2G44890	CYP704A1	No blast hit	-	-	Cre01.g003850.t1.3	8.0E-45	2.0E-50
		AT2G45970	CYP86A8	kfl00171_0160	4.0E-138	7.0E-138	No blast hit	-	-
		AT1G69500	CYP704B1	No definite counterpart		-	No blast hit	-	-
	HOTHEAD (ω-OH fatty acyl dehydrogenase)	AT1G72970	HTH/EDA17	kfl00011_0020	2.0E-83	1.0E-83	Cre12.g514200.t1.2	4.0E-32	4.0E-32
Polymer synthesis	Cutin synthase-like protein (homolog of CD1)	AT3G04290	AtCUS1/ LTL1	Kfl00011_0050	3.0E-40	5.0E-40	No blast hit	-	-
	Defective in cuticular ridges	AT5G23940	DCR	No blast hit	-	-	No blast hit	-	-
	Hydroxycinnamoyl-CoA shikimate/ quinate hydroxycinnamoyl transferase	AT5G48930	HCT	kfl00513_0110	1.0E-50	2.0E-50	No blast hit	-	-
GDSL-motif lipase	GDSL-motif lipiase5	AT1G53920	GLIP5	kfl00300_0110	4.0E-59	5.0E-59	No blast hit	-	-
HXXXD-type acyltransferase family protein	Acetyl CoA:(z)-3-hexen-1-ol acetyltransferase	AT3G03480	CHAT	No blast hit	-	-	No blast hit	-	-
	Spermidine disinapoyl acyltransferase	AT2G23510	SDT	No blast hit	-	-	No blast hit	-	-
